# A point-mutation in the C-domain of CMP-sialic acid synthetase leads to lethality of medaka due to protein insolubility

**DOI:** 10.1038/s41598-021-01715-3

**Published:** 2021-12-01

**Authors:** Di Wu, Hiromu Arakawa, Akiko Fujita, Hisashi Hashimoto, Masahiko Hibi, Kiyoshi Naruse, Yasuhiro Kamei, Chihiro Sato, Ken Kitajima

**Affiliations:** 1 Institute of Glyco-Core Research, Nagoya University, Chikusa, Nagoya 464-8601 Japan; 2 Bioscience and Biotechnology Center, and Graduate School of Bioagricultural Sciences, Nagoya University, Chikusa, Nagoya 464-8601 Japan; 3 Graduate School of Science, Nagoya University, Chikusa, Nagoya 464-8601 Japan; 4 National Institute of Basic Biology, Nishigonaka 38, Myodaiji, Okazaki 444-8585 Japan; 5 Department of Basic Biology, School of Life Science, The Graduate University for Advanced Studies, Shonan Village, Hayama, Kanagawa 240-0193 Japan

**Keywords:** Glycobiology, Medaka, Enzymes, Glycobiology

## Abstract

Vertebrate CMP-sialic acid synthetase (CSS), which catalyzes the synthesis of CMP-sialic acid (CMP-Sia), consists of a 28 kDa-N-domain and a 20 kDa-C-domain. The N-domain is known to be a catalytic domain; however, the significance of the C-domain still remains unknown. To elucidate the function of the C-domain at the organism level, we screened the medaka TILLING library and obtained medaka with non-synonymous mutations (t911a), or single amino acid substitutions of CSS, L304Q, in the C-domain. Prominently, most L304Q medaka was lethal within 19 days post-fertilization (dpf). L304Q young fry displayed free Sia accumulation, and impairment of sialylation, up to 8 dpf. At 8 dpf, a marked abnormality in ventricular contraction and skeletal myogenesis was observed. To gain insight into the mechanism of L304Q-induced abnormalities, L304Q was biochemically characterized. Although bacterially expressed soluble L304Q and WT showed the similar *V*_max_/*K*_m_ values, very few soluble L304Q was detected when expressed in CHO cells in sharp contrast to the WT. Additionally, the thermostability of various mutations of L304 greatly decreased, except for WT and L304I. These results suggest that L304 is important for the stability of CSS, and that an appropriate level of expression of soluble CSS is significant for animal survival.

## Introduction

Sialic acids (Sias) are a group of nine-carbon sugars located at the termini of glycoproteins and glycolipids on cell surfaces. In addition to their presence on outermost positions on cell surfaces, Sias have many unique properties such as negative charges, diverse modes of linkages, and large repertoires of natural modifications. The expression of Sias is also regulated, both developmentally and organically^1–5^. For example, Sias are highly expressed in podocalyxin in the mammalian kidney, indicating the formation of glomerular filtration^6^. Large amounts of polysialic acid (polySia) residues are present in the developing mammalian brain. PolySia is not only known to inhibit the homophilic binding of neural cell adhesion molecules (NCAMs)^7,8^, but also retain various physiologically important molecules to regulate their functions^9–12^. However, the significance of the spatiotemporal expression of Sia at the organism level, in animals, remains largely unknown.

CMP-sialic acid synthetase (CSS or CMAS) is a key enzyme for the expression of sialoglycoconjugates on the cell surface because it activates free Sia, converting it to CMP-Sia, which is the only donor substrate for all sialyltransferases^13–15^. The vertebrate CSS contains two domains: the N-domain and C-domain. Although the N-domain is highly conserved among most organisms, the C-domain is known to exist only in vertebrates and some types of bacteria^16–19^. The C-domain of mouse CSS (mCSS) is not a catalytic domain as it maintains enzymatic activity without the C-domain^20^. Although the crystal structure of the C-domain of mCSS revealed that the C-domain forms a tetrameric structure, which appears to affect overall oligomerization state of mCSS^20^, its role in structural properties or biological function remains unclear.

To gain new insight into the biological significance of the C-domain of vertebrate CSS at the animal level, we chose to use medaka (*Oryzias latipes*) as a vertebrate model. Medaka are useful for studying the biological function of CSS and Sia-glycoconjugates for the following reasons: medaka contain genes for enzymes and transporters that are necessary for expression of Sia, including *cmas*, a gene for CSS. They serve as ideal vertebrate models because of their ease of handling, large numbers of progeny per generation, and in particular, their translucent embryos, which enable easy observations^21^. Furthermore, their stages of normal development have been well studied and described^22^. The greatest advantage, however, is that most reverse genetics approaches are applicable.

In order to determine whether the C-domain of CSS plays a role at the organism level, we searched for medaka models with point mutations in the C-domain of the *cmas* through screening of the medaka Targeting-Induced Local Lesion IN Genome (TILLING) library^23^, and found a medaka strain with a single mutation in the C-domain of CSS (L304Q), which was lethal to young fry by three weeks old. Together with the severe phenotypes of the L304Q medaka, an induction of instability of the CSS molecule by this mutation demonstrated the significance of the C-domain at the organism and molecule levels.

## Results

### A point-mutation in the C-domain of CSS was lethal in medaka fish

To study the biological function of the C-domain of CSS using medaka as an animal model, we screened the medaka TILLING library with 5,760 mutant medaka strains^23^ The medaka *cmas* (a gene for CSS) is located on chromosome 18, and encodes 459 amino acids (ENSORLG00000018421). The N-domain of medaka CSS (mdkCSS) contains five conserved motifs with high similarity to other vertebrate CSSs, while the C-domain of mdkCSS shows less homology to the C-terminal domains of CSS in other organisms (human, mouse, and rainbow trout) (Supp_FigS1). To identify medaka with a mutation in the C-domain, we targeted exons 6–7 of the *cmas* gene, screening the TILLING library by the high-resolution melting curve (HRM) assay^24^. This was followed by re-sequencing of the PCR fragments. Finally, only L304, considered to be a missense mutation, was found.

**Figure 1:**
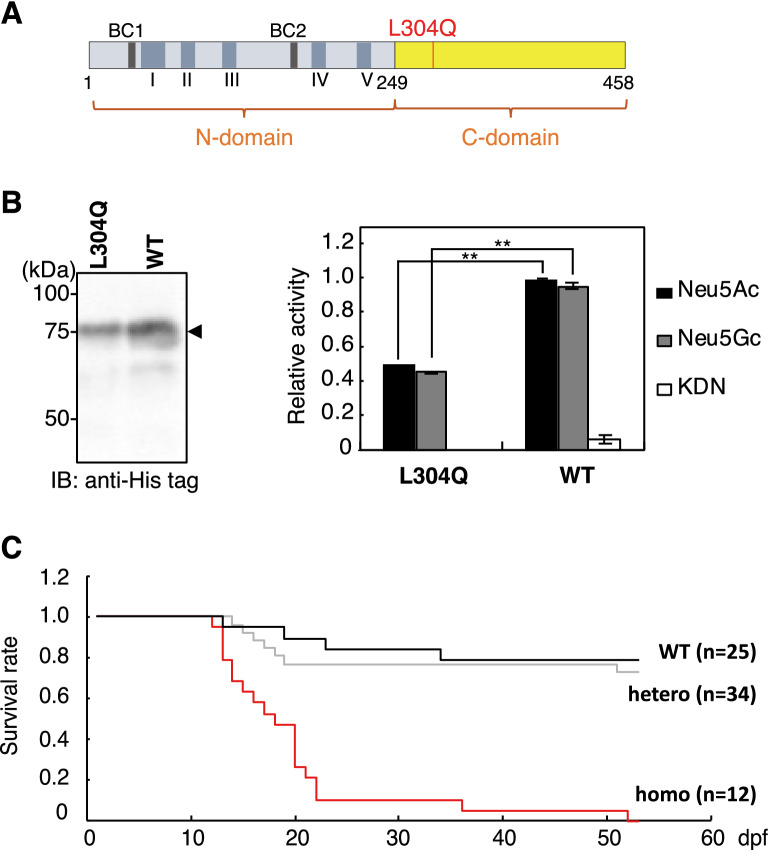
Properties of L304Q mdkCSS and survival curve of L304Q homo medaka. (**A**) Schematic drawing of L304Q mdkCSS. The position of L304Q site is shown by red bar. I ~ V, the evolutionarily conserved amino acid sequence motifs; BC1 and BC2 represent basic amino acid clusters that may be functional as nuclear localization signals. (**B**) Enzyme activity of L304Q mdkCSS. *Left*, western blotting of the recombinant L304Q and WT using anti-His antibodies was performed for quantifying the enzyme amount. *Right*, the relative specific activity of L304Q and WT in relation to Neu5Ac, Neu5Gc and KDN. The specific activity of WT in relation to Neu5Ac was set to 1. The bars represent standard deviation from three independent experiments. (**C**) Survival curve of medaka with the L304Q mutation in the *CSS* gene. WT, hetero, and L304Q homo medaka were derived from in-crossing of the same L304Q hetero parents. The numbers (n) of fish examined are shown in the parentheses. **indicates p < 0.005. Full-length blots for (**B**) are presented in Supp_FigS7.

In order to determine the in vitro activity of this missense mutant mdkCSS from the TILLING library, recombinant L304Q mdkCSS (L304Q) protein and wild-type mdkCSS (WT) protein (Fig. 1A) were expressed in *Escherichia coli*. The recombinant CSS protein showed a main band at 75 kDa (Fig. 1B, *left*), which was the molecular mass of mdkCSS with His-tag, S-tag, and thioredoxin-tag in front of the N-terminus of mdkCSS. The in vitro activities of crude mdkCSS proteins were performed with excess substrates, as described in the *Methods* section. As shown in Fig. 1B, *right*, L304Q had lower enzymatic activity, in relation to Neu5Ac, Neu5Gc, and KDN, than WT.

After the background mutations in L304Q medaka fish were removed by artificial insemination^25^ and outcrossed with wild-type medaka fish (WT) for more than five generations, mdkCSS^304Q/Q^ (homo), mdkCSS^304L/Q^ (hetero), and mdkCSS^304L/L^ (WT) medaka were obtained by crossing heterozygous adult medaka (https://shigen.nig.ac.jp/medaka/download/strain/NBRP_TILLING_en.pdf). Genotype of each medaka was determined by HRM assay and sequencing analysis (Supp_FigS2). The homo medaka could hatch at 9 dpf and appeared to grow normally until 14 dpf. After that, however, homo medaka fry began to be dead (Fig. 1C). The median lifespan of homo medaka was 19 dpf, which is the 1st fry stage in medaka development^22^, and very few homo medaka could survive until 50 dpf (Fig. 1C). On the other hand, the WT and hetero medaka fry could survive normally until adult fish, although 20% of them were lethal during early developmental stage (Fig. 1C). In any experiments below, the homo, hetero, and WT medaka that were obtained by crossing the same heterozygous adult after at least six generations were used. Therefore, we can safely say that they had strictly the same genetic background except for the point-mutation in question.

### Sialylation was reduced in L304Q homo medaka

**Figure 2:**
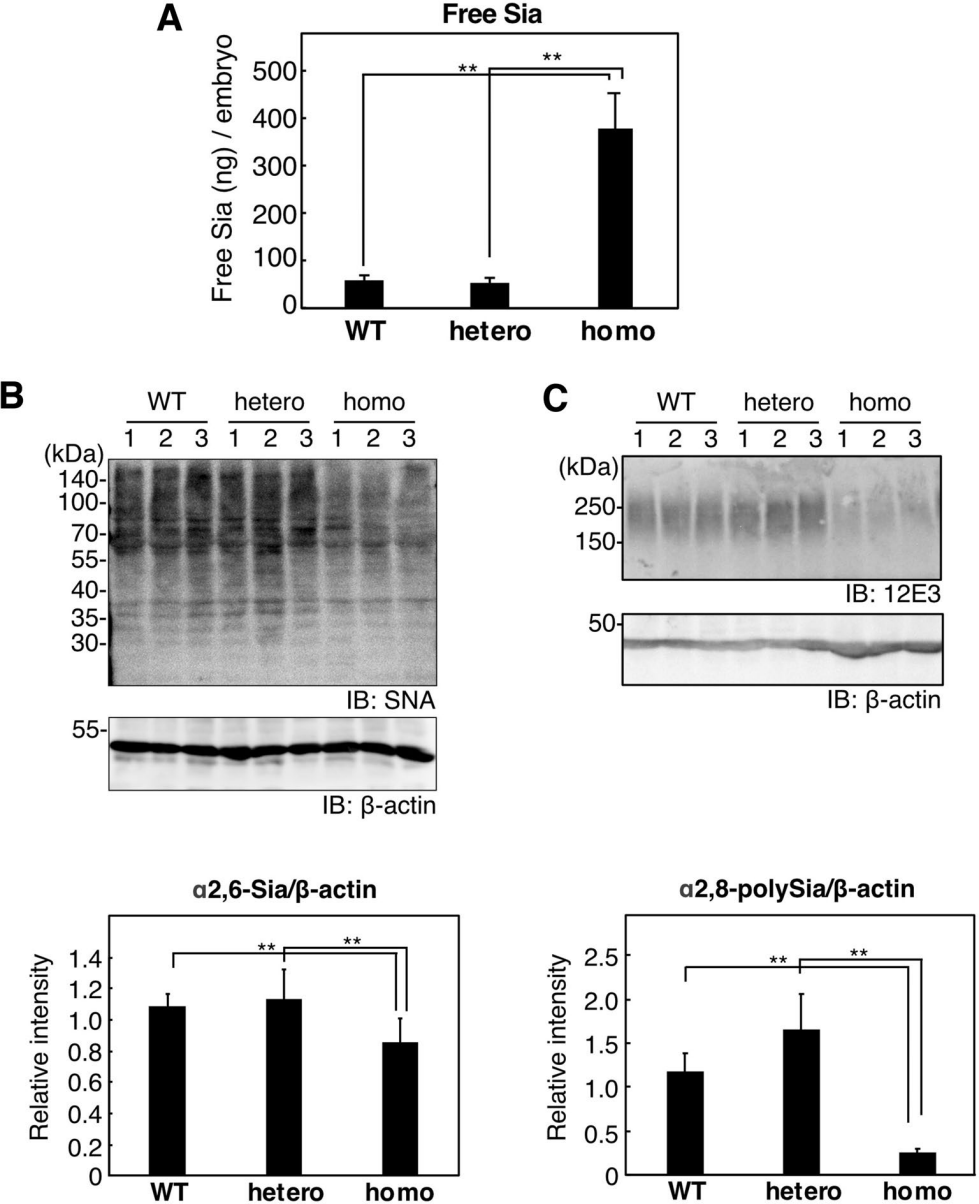
Free and bound Sia in the whole lysate of WT, hetero, and homo medaka of L304Q at 8 dpf. (**A**) The amount of free Neu5Ac in the lysate was quantified by the fluorometric HPLC analysis, as described in *Experimentnal Procedures*. The bars represent standard deviations from three independent experiments. (**B**) The α2,6-Sia epitope evaluated by a lectin blotting with SNA and (**C**) the α2,8-polySia epitope evaluated by the western blotting with anti-polySia antibody (12E3). The lysate was applied to SDS-PAGE, followed by the SNA lectin-staining (**B**, *upper panel*) and 12E3-immuostaining (**C**, *upper panel*), respectively. Lanes 1–3 stand for homogenates from three independent fry. Immunoblotting of β-actin was used as a loading control (**B**,**C**, *middle panels*). The relative amounts of the Sia epitope are also shown (*lower panels*). The bars represent standard deviations from the data obtained from three independent fish. **indicates p < 0.005. Full-length blots for (**B**,**C**) are presented in Supp_FigS8 and Supp_FigS9, respectively.

To understand whether the L304Q mutation in the C-domain of mdkCSS impaired enzymatic activity in vivo, the change in free Sia (a substrate of CSS) in L304Q homo medaka at 8 dpf was quantified by HPLC (Fig. 2A). The quantity of free Neu5Ac accumulated in homo medaka was 50-fold higher than that in hetero and WT medaka fry. Furthermore, α2,6-sialic acid and α2,8-linked polySia epitopes in L304Q medaka fry were quantified by lectin-blotting with SNA lectin and western blotting using 12E3 antibody, respectively (Fig. 2B,C). The amount of both epitopes in homo medaka fry decreased after 8 dpf. It should be noted that α2,3-sialic acid epitopes were not obviously detected at 14 dpf, compared with the other two epitopes, as probed by MAA-lectin blotting (Supp_FigS3). The MAA epitopes were detected in all the homo, hetero, and WT embryos at 8 dpf, because they came from yolk materials that are known to be maternally derived, but not zygotically expressed (Supp_FigS3).

### Cardiomyopathy was observed in L304Q homo medaka

**Figure 3:**
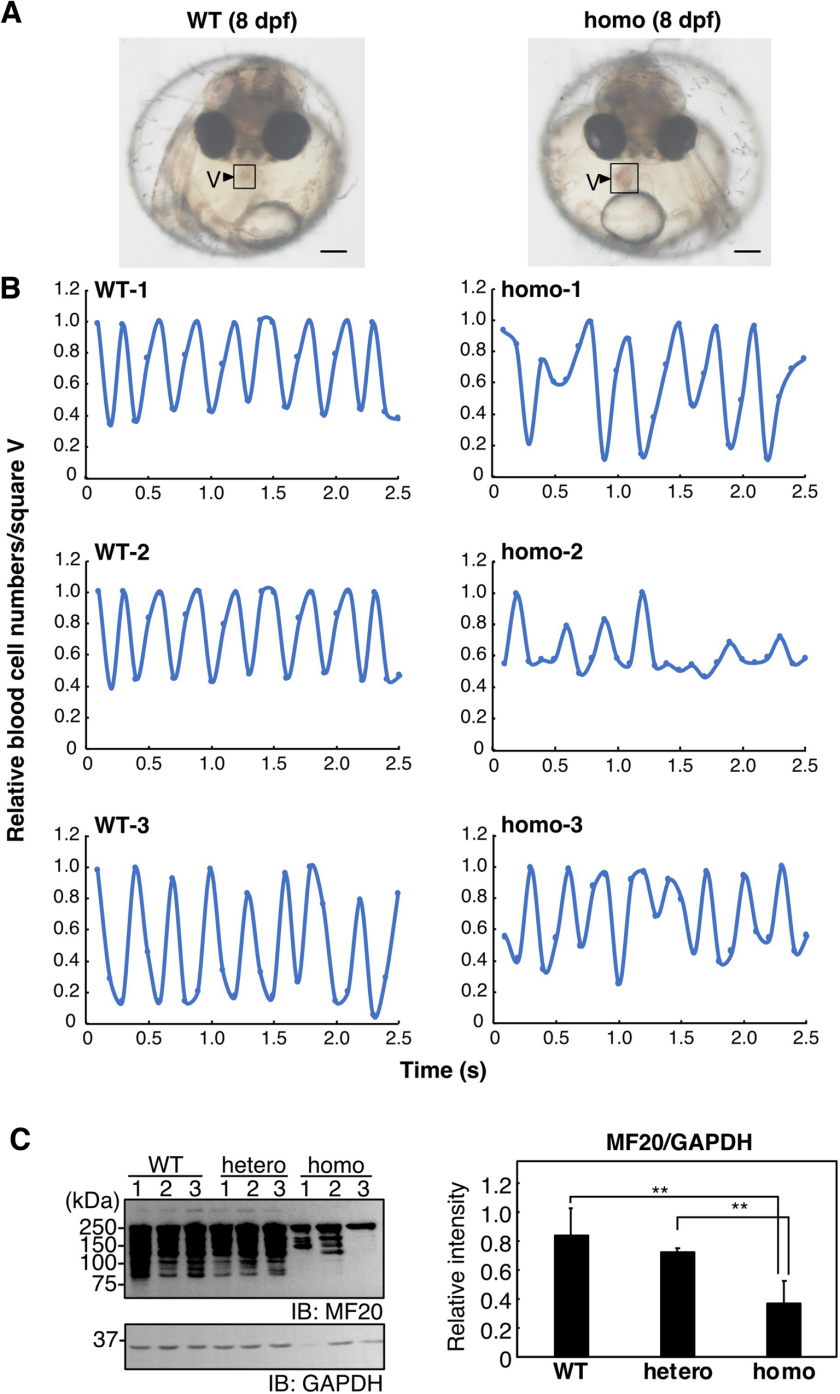
Abnormalities of heart development and function of L304Q homo medaka. (**A**) Morphological image of WT (*left*) and L304Q homo medaka (*right*) at 8 dpf. The scale bars indicate 200 mm. The square marked by V shows the ventricle of heart. (**B**) Periodic characteristics of ventricle contraction in WT and L304Q homo medaka at 8 dpf. Periodical changes of the blood cell density in the ventricle at the square V (in **A**) were measured as those of the light and shade intensity using ImageJ. The data from three individual medaka for WT and homo are shown. (**C**) The expression of myosin heavy chain (MHC) in WT and L304Q homo medaka at 14 dpf. The lysate was analyzed by the SDS-PAGE/western blotting using MF20 antibody, which recognizes the heavy chain of myosin II and have been used to highlight the myocardium of medaka (26)(*left upper*). GAPDH was also detected by anti-GAPDH antibody as the loading control (*left lower*). The MHC components were decreased in the homo medaka, which may indicate cardiomyopathy. **indicates p < 0.005. Full-length blots for (**C**) are presented in Supp_FigS10.

It has been shown that aberrant sialylation causes dilated cardiomyopathy and myopathy^26^. Therefore, the heart development of L304Q homo medaka was observed from 0 to 9 dpf (the hatching stage). L304Q homo embryos appeared to be normal until 6 dpf (the heart development stage); however, from 7 dpf (pericardial cavity formation stage), there often observed such a phenomenon that blood could not completely be pumped out from their ventricles. One contraction out of three was often incomplete (Fig. 3B, homo-1 and 3), which led to the clogging of blood in the ventricle for a longer time in homo embryos than in WT subjects. When the patterns of ventricular contraction and expansion were monitored, ventricular arrhythmia was also observed in homo medaka (Fig. 3B). To confirm the protein level of myosin heavy chain (MHC) in medaka, western blotting using MF20 monoclonal antibodies, which recognizes the heavy chain of myosin II and have been used to highlight the myocardium of medaka at early developmental stage, was performed^27,28^ (Fig. 3C), showing that the expression level of MHC protein in homo medaka fry at 14 dpf was much lower than that in WT. These results indicated that cardiomyopathy occurred in the homo fry, which may explain the lethality of the homo medaka.

**Figure 4:**
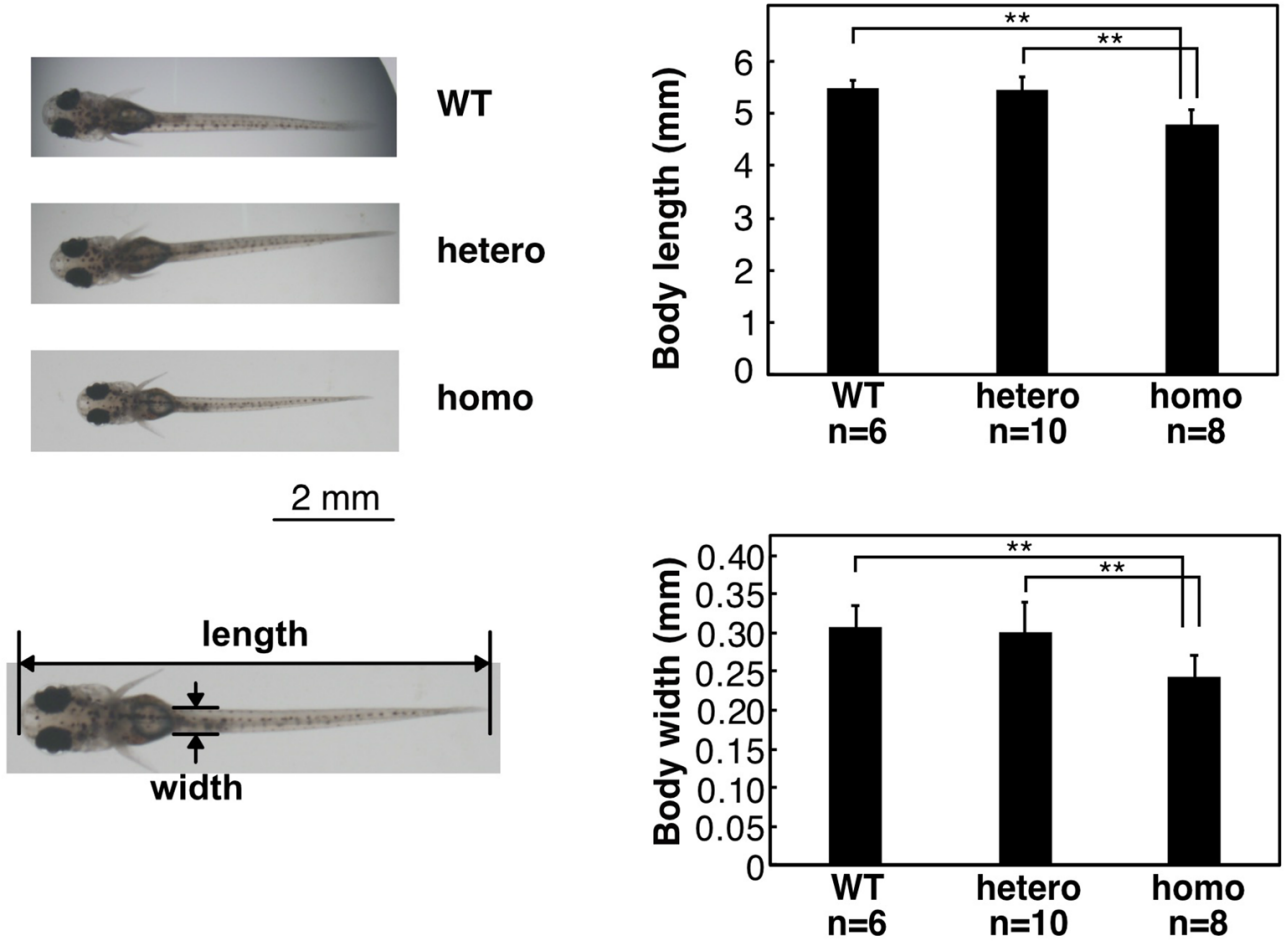
Body length of L304Q medaka at 14 dpf. Images of WT, hetero, and L304Q homo medaka fry at 14 dpf were observed under the microscope (*left*). The scale bars indicate 2 mm. The body lengths of medaka fry at 14 dpf were measured and the data are summarized in a bar graph (*right*). n, the number of fry measured. The bars represent standard deviations obtained for the indicated numbers of fry. **indicates p < 0.005.

To see how skeletal myogenesis was going, the body size was compared at 14 dpf (Fig. 4). The body length and body width of homo fry were significantly shorter than those of WT and hetero fry. However, no obvious behavioral anomaly was observed. These results suggest that muscle development might be impaired, though not seriously, in homo fry.

### Comparison of enzyme kinetics of L304Q and WT mdkCSS

**Figure 5:**
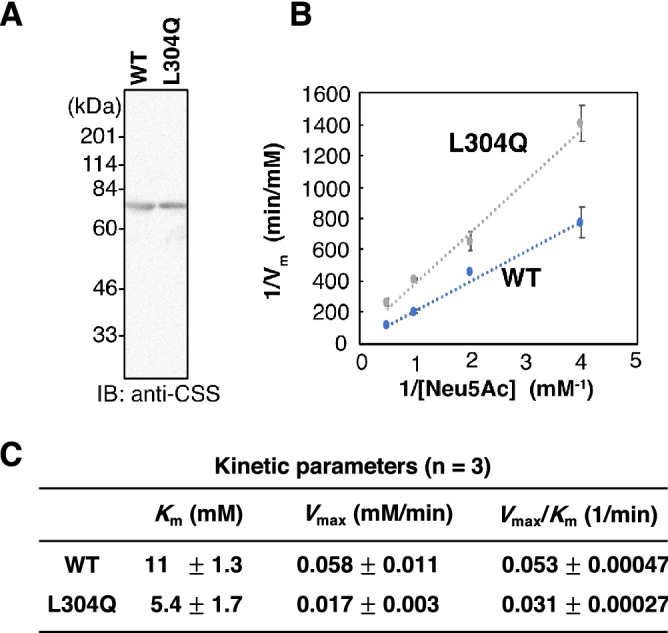
Kinetic analysis of WT and L304Q mdkCSS. (**A**) Purified recombinant enzymes used for the kinetic analysis. SDS-PAGE/CBB staining (*left*). Western blotting using the anti-mdkCSS antibodies (*right*). Full-length blots for A are presented in Supp_FigS11. (**B**) Lineweaver–Burk plots for the synthesis of CMP-Neu5Ac by mdkCSSs. (**C**) Kinetic parameters. *K*_m_, *V*_max_, and V_max_/*K*_m_ values are expressed as average values ± standard deviations from three independent experiments (n = 3).

The lethality of L304Q homo medaka led us to investigate effects of the L304Q mutation in the C-domain of CSS on enzyme properties. First, kinetic analysis of L304Q and WT mdkCSS was performed using purified soluble proteins. Recombinant mdkCSS proteins were expressed in *E. coli* and purified by affinity chromatography on a Ni^2+^ chelating column. The purified mdkCSS proteins were then quantified by western blotting using an anti-mdkCSS antibody, and showed a main band at 75 kDa (Fig. 5A). For kinetic analysis, each reaction was performed at 25 °C with for 15 min using 0.2 µg of the purified recombinant mdkCSS protein, because these conditions allowed a linear increase in the formation of products. Neu5Ac was used as the substrate from 0 to 4 mM, and CTP was used at a saturating concentration of 5 mM. Based on the Lineweaver–Burk plot (Fig. 5B), the *K*_*m*_ values indicated that the L304Q exhibited twice as high affinity for Neu5Ac as WT, and the *V*_*max*_ values showed that the L304Q had three times slower reaction velocity than WT. The *V*_*max*_*/K*_*m*_ values were thus determined as 0.031 min^-1^ for L304Q and 0.053 min^-1^ for WT (Fig. 5C), indicating that the efficiency of producing CMP-Sia of L304Q was 58% of that of WT. These results indicate that the L304Q mutation lowers the enzymatic activity of mdkCSS, but this reduction effect is not severe under conditions for kinetic analysis, *i.e.*, in a short-time (15 min) incubation at 25 °C.

### L304Q mutation in the C-domain of mdkCSS lowered the solubility in vivo

**Figure 6:**
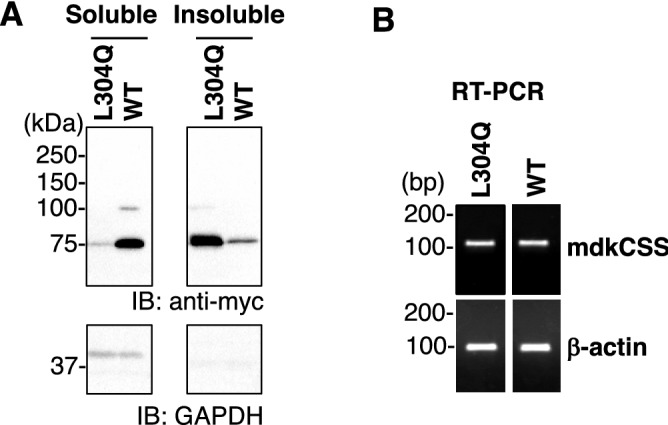
Property of WT and L304Q mdkCSS expressed in CHO cells. (**A**) Distribution of mdkCSS in the soluble and insoluble fractions. The myc-tagged WT and L304Q mdkCSS were transiently expressed in CHO cells, and their homogenates were separated into the soluble and insoluble fractions by the high speed centrifugation, followed by western blotting with the anti-myc antibody. Immunostaining with anti-GAPDH was performed as the loading control for soluble fraction. Note that the GAPDH bands were not detected in the insoluble fraction, which is reasonable because that GAPDH is a soluble protein. The band at 75 kDa stands for mdkCSS. (**B**) Expression level of the mRNA for mdkCSS. The transfected CHO cells expressing the myc-tagged WT and L304Q mdkCSS were analyzed by RT-PCR using the specific primers. The expression level of β-actin was used as the loading control. Full-length blots/gels for A and B are presented in Supp_FigS12 and Supp_FigS13, respectively.

In the course of preparation of the recombinant L304Q CSS using *E. coli*, we noticed that an yield of soluble protein for L304Q was significantly lower than that for WT, suggesting that L304Q is less soluble than WT. To understand whether the L304Q mutation impaired the solubility of CSS in vivo, myc-tagged mdkCSS proteins were transiently expressed in CHO cells. The CSS protein level was quantified by western blotting using anti-myc antibody (Fig. 6A) and the mRNA levels of mdkCSSs were quantified by RT-PCR (Fig. 6B). The mRNA levels of L304Q and WT were at the same level (Fig. 6B). On the other hand, the protein levels of L304Q and WT detected in the soluble and insoluble fractions were greatly different. For L304Q, the protein was mainly detected in the insoluble, but not soluble fraction; in contrast, for WT, mainly in the soluble, but not insoluble fraction. This was notably different between L304Q and WT. These results suggest that the L304Q CSS protein easily becomes insoluble after translation when expressed in CHO cells.

We then sought to investigate the intracellular localization of L304Q and WT in CHO cells. We found that unusually round-shaped cells were prominently increased in L304Q-expressing cells, while such abnormal-shape cells were very few in WT-expressing cells (Supp_FigS4A). The proportions of their cell numbers were 25% and 2% for L304Q- and WT-expressing cells, respectively. The abnormal-shape cells did not proliferate and appeared to be finally dead. On the other hand, the normal-shape cells showed much the same intracellular localization profiles for both L304Q- and WT-expressing cells (Supp_FigS4B). It is thus suggesed that the L304Q mutation has no effect on the nuclear-cytosolic localization property of CSS, and that L304Q CSS, once expressed, gradually induces protein aggregation to form the insoluble protein aggregates (Fig. 6A). The protein aggregates might cause the cell shape changes to abnormal-shape cells. However, further studies are necessary to gain further insight into the L304Q CSS-driven intracellular events.

### The non-polar amino acid at position 304 of the C-domain was critical for thermostability of the activity

**Figure 7:**
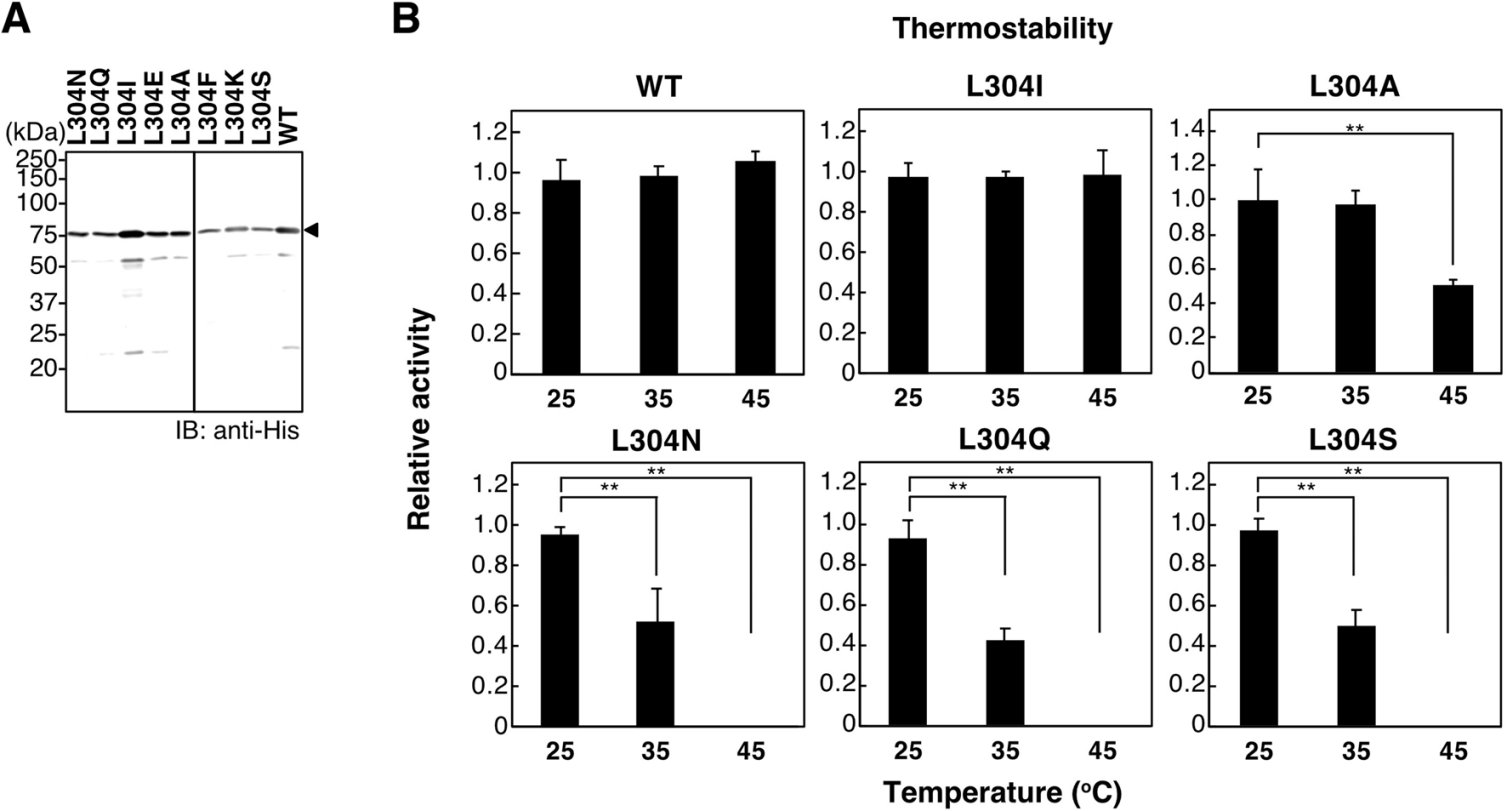
In vitro properties of the single amino acid-substituted mutants at L304. (**A**) Western blotting of the recombinant mdkCSS mutants, L304N, L304Q, l304I, L304E, L304A, L304F, L304K, L304S, and WT with the anti-His antibody. The soluble forms were obtained from all the recombinant proteins expressed in *E. coli*. Full-length blots for A are presented in Supp_FigS14. (**B**) Thermostability of the L-304 mutants and WT of mdkCSS. Recombinant CSS proteins were pre-heated at 25, 35, or 45 °C for 15 min and measured for in vitro CSS activity. Relative in vitro activity of CSS was obtained as the amount of produced CMP-Neu5Ac divided by the intensity of the recombinant protein, and the value for mdkCSS without pre-heating (*i.e.*, 25 °C) was set to 1.0. The bars represent standard deviations from three independent experiments. **indicates p < 0.005.

To gain more insight into the insolubility of L304Q CSS, Leu-304 was mutated into various amino acids using the Quick Change Lightning Site-Directed Mutagenesis method, and examined for the thermostability of the activity in vitro. Each His-tagged mdkCSS protein was expressed in *E. coli* cells, and its protein levels were analyzed by western blotting using anti-His antibodies (Fig. 7A). All the recombinant mdkCSS proteins were successfully prepared as a soluble enzyme, although their yields in the soluble fraction were different from each other. They were then pre-heated at 25, 35, and 45 °C for 15 min before the in vitro activity toward Neu5Ac was measured (Fig. 7B). When Leu-304 changed to a non-polar amino acid (Ile) the CSS activity remained the same as that in WT at any pre-heat temperatures tested. When Leu-304 changed to Ala, which contains a shorter hydrophobic side chain than Leu, the enzyme lost half of its activity once pre-heated at 45 °C for 15 min. When Leu-304 was changed to polar amino acids (Gln, Asn, and Ser), CSS completely lost its activity once it was pre-heated at 45 °C for 15 min (Fig. 7B). It was shown that the non-polar amino acid at the 304 position of the C-domain of mdkCSS was essential for maintaining the thermostability of the enzyme activity.

The thermostability of protein itself was further examined for the L304Q and WT CSS by the protein melting assay. The melting curves (Supp_FigS5) revealed that the melting points (Tm) for L304Q and WT CSS were 48.6 and 45.1 °C, respectively, indicating that the L304Q protein is slightly more thermo-stable than the WT. However, this Tm difference may not be significant, because the L304Q CSS activity prominently decreases at 35–45 °C below the Tm, while the WT CSS activity remains unchanged by sharp contrast. It might thus be considered that the L304Q mutation in the C-domain induces such structural changes that decrease the catalytic activity of the N-domain without changing the protein stability so much.

### L304Q and WT medaka CSS had similar oligomerization states

It has been shown that the C-domain is involved in oligomerization in mouse CSS^20^. To investigate if the oligomerization state of the L304Q CSS was different than the WT CSS, the native-PAGE experiments were performed (Supp_FigS6). The L304Q and WT CSSs gave much the same profiles with a major component at around 240 kDa on the native-PAGE, suggesting that the oligomerization status of the soluble L304Q CSS was not so different than the WT CSS.

## Discussion

The L304Q medaka model provides the first insight into the biological significance of the C-domain whose presence is unique to vertebrate CSSs. Namely, the L304Q medaka show the 50% lethality at 19 dpf (Fig. 1C) and an impact of the mutation is serious in the survival of this organism. The accumulation of free Sia and the concomitant reduction of α2,6-Sia and α2,8-linked polySia epitopes (Fig. 2) indicate that L304Q medaka lose all CSS activity in vivo. This phenomenon appears to be contradictory to our kinetic data showing that the L304Q mutation has no severe effect on the enzymatic activity at least under conditions used for kinetic analysis, *i.e.*, 15 min incubation at 25 °C (Fig. 5). Interestingly, however, when the L304Q protein is expressed in CHO cells at 37 °C, the L304Q protein is nearly absent in a soluble fraction, and instead accumulated in an insoluble fraction, while, by sharp contrast, WT protein is exclusively recovered in a soluble fraction (Fig. 6). Thus, the L304Q mutation brings about the insolubility under in vivo situations. Notably, in the L304Q-expressing cells, the abnormal-shape cell population prominently increases, probably because the insoluble protein aggregates might facilitate the cell shape changes through an unknown mechanism.

**Figure 8:**
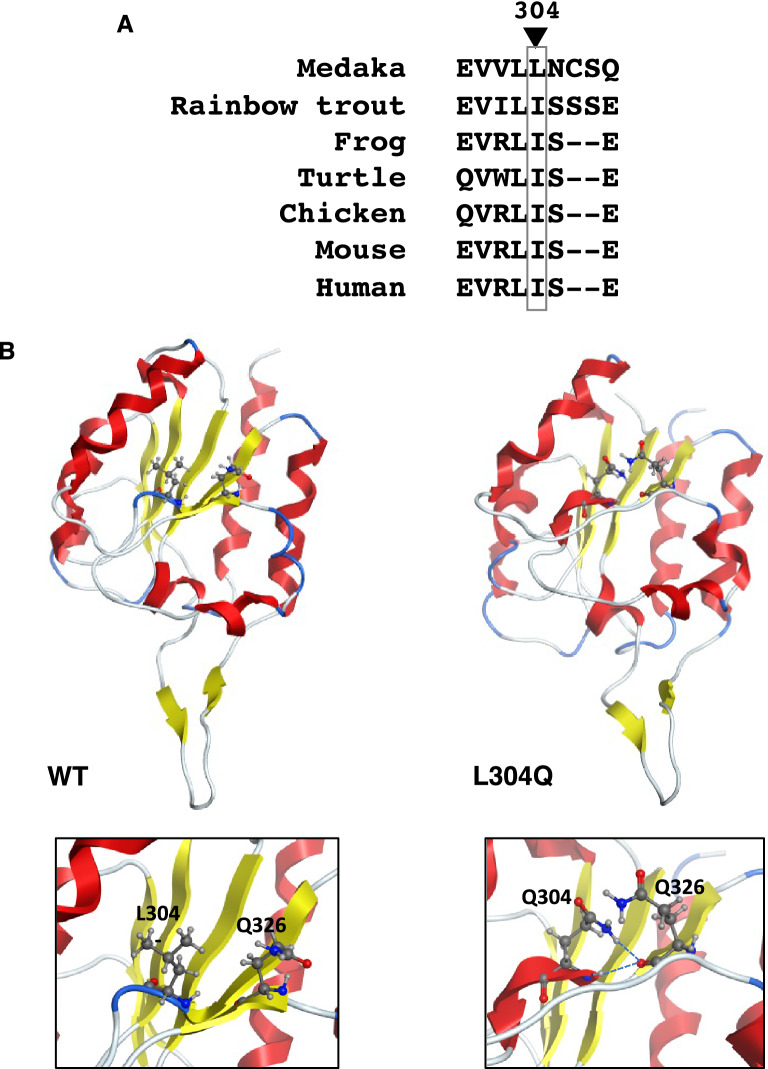
Molecular modeling for WT and L304Q mdkCSS. (**A**) Comparison of the local amino acid sequence around L-304 residue among vertebrates. Medaka, O*ryzias latipes*; Rainbow trout, *Oncorhinchus mykiss*; Frog, Xenopus tropicalis; Turtle, *Mauremys reevesii*; Chicken, *Gullus gullus*; Mouse, *Mus musculus*; Human, *Homo sapiens*. (**B**) Molecular modeling of the C-domains of WT and L304Q. These models are constructed by the MOE homology modeling based on the crystal structure of the C-domain of mouse CSS (PDB: 3EWI). Magnified views around L-304 residue are shown at lower panels. Two hydrogen bonds newly appear between H of NH group of the Q304 side chain and O of carbonyl group of the Q326 backbone, and between H of NH group of the Q304 backbone and O of carbonyl group of the Q326 backbone. No such interaction is not observed in WT.

To understand how this point-mutation in the C-domain of mdkCSS changes the solubility of the enzyme, the thermostability of the CSS activity was investigated using a series of L304 substituted CSS mutants, showing that the non-polar long-side chain amino acids are critical for thermostability of the CSS activity (Fig. 7). Obviously, the L304Q CSS activity is highly temperature-sensitive more than the WT. However, the Tm values of the CSS proteins are much the same between them, i.e. 45–48 °C, with a slightly higher Tm for L304Q than WT. Therefore, it is difficult to conclude that the thermal instability of the L304Q CSS is related to its insoluble property in cell. It is rather considered that the L304Q might form those particular structures not only that decrease the catalytic activity of the N-domain at 35–45 °C, but also that allow undesired complex formation, leading to the easy accumulation in the insoluble fraction when expressed in CHO cells. Based on the results of the native-PAGE of purified CSSs (Supp_FigS6), it appears that the L304Q mutation does not seriously affect their homo-oligomer formation, as far as L304Q CSS is purified as a soluble form. It is thus suggested that the undesired complex of the L304Q CSS might be formed with other molecules than the CSS. It is notable to point out that Leu/Ile at this position, in addition to the adjacent conserved Leu residue, is highly conserved in other vertebrate CSSs (Fig. 8A), which suggest the importance of this Leu/Ile throughout evolution of the vertebrate CSS. Furthermore, the 3D structures of L304Q and WT mdkCSS are constructed from the crystal structure of the C-domain of mouse CSS^20^ using MOE homology modeling (Fig. 8B). Leu-304 is located in the β4-sheet, which is situated in the interior of the C-domain of CSS, and neither at the boundary surface of the C-domain oligomer^20^. When Leu-304 is mutated to Gln, H’s of NH groups at both of the side chain and the backbone of the Gln-304 residue form extra hydrogen bonds with O of the carbonyl group of backbone Gln-326 residue (Fig, 8B, magnified image). The formation of the extra hydrogen bonds may explain the slightly higher Tm of L304Q compared with WT. The amino acid sequence of the C-domain is homologous to a bacterial sugar phosphatase of the haloacid dehalogenase (HAD) superfamily that catalyzes carbonyl or phosphoryl group transfer reactions. However, neither mCSS nor its C-domain was shown to exhibit phosphatase activity in vitro^20^. The 3D structure of mCSS C-domain has been described as a functional platform for the dimerization of the catalytic domain; that is, the N-domain of CSS^20^. Taken together, it remains to be clarified how this point-mutation at the 304 position of the C-domain is related to the overall structural change of whole molecule leading to the enzymatic insolubility; however, this is the first structure–activity relationship study of the C-domain, which suggests the importance of the C-domain intactness.

The lethality of L304Q medaka, together with the observation of the accumulation of free Sia and the extensive reduction of α2,6-Sia and polySia epitopes from 8 dpf, indicates that sialylation is essential for the development of medaka. It has been shown that mutations in Sia metabolism-related genes lead to various human diseases, such as muscular dystrophy, skeletal dysplasia, and intellectual developmental disorders^29–33^. Cardiovascular disorders have also been reported sporadically in patients with the UDP-GlcNAc 2-epimerase/ManNAc kinase (GNE) myopathy and SLC7A4 deficiency, and the cardiac phenotype has also been observed in the *N*-acetylneuraminate-pyruvate lyase gene (*npl*) morphant zebrafish^34^. Hyposialylation often causes enlargement of ventricles and weakness in ventricular contraction^26^. As shown in this study, the amount of myosin heavy chain in the hearts of L304Q mutant medaka is significantly reduced at 14 dpf (Fig. 3), indicating that cardiomyocyte production is affected by a sialylation level. Furthermore, the body length and width of L304Q homo medaka fry were also obviously smaller than WT and hetero fry at 14 dpf, indicating that skeletal myopathy also occurs in L304Q homo medaka fry without any behavioral anomalies. The abnormalities of heart motions, such as a clogging of blood in the ventricle and ventricular arrhythmia, observed in the L304Q homo medaka at 8 dpf may also be a sign of the impaired heart development. However, mechanisms by which the reduced sialylation level in L304Q mutant leads to the functional impairment of heart remain to be clarified. For other organs than heart, it is known that sialylation is important for podocyte maturation in mice, with altered sialylation impairing podocyte maturation^35^. However, the tissue morphology of kidney remains unchanged in WT and L304Q homo medaka fry, although the α2,6-Sia epitope disappears in L304Q homo, compared with that in WT medaka fry (data not shown). Further studies on kidney function are necessary in medaka.

## Methods

### Materials

Neu5Ac, CMP-Neu5Ac, isopropyl-1-β-D-galactopyranoside (IPTG), aprotinin, and leupeptin were from nacalai tesque (Kyoto, Japan). Neu5Gc was from Tokyo Chemical Industry Co. (Japan). KDN was prepared as described previously^36^. 1,2-dimethylenedioxybenzen (DMB) was purchased from Dojindo Molecular Technologies, Inc. (Kumamoto, Japan). Pre-stained Mw marker was obtained from Bio-Rad (Hercules, CA). Enhanced chemiluminescence (ECL) reagents and protein G-Sepharose resins were purchased from Amersham Biosciences (Piscataway, NJ). PVDF membrane (Immobilon P) was a product of Millipore (Bedford, MA). Molecular marker was purchased from Sigma (St. Louis, MO) and BIO-RAD. Ni–NTA-Agarose was purchased from QIAGEN (Valencia, CA). The recombinant enterokinase was a product of Novagen (Tokyo, Japan). An anti-polySia antibody, 12E3, which recognizes the oligo/polyNeu5Ac structure (DP ≥ 5)^37^, was generously provided by Dr. Tatsunori Seki (Tokyo Medical University, Tokyo, Japan). Pure *Sambucus nigra* lectin (SNA), which recognizes Siaα2 → 6Gal or GalNAc, were purchased from EY Laboratories, INC. (San Mateo, CA). Mouse anti-lectin antiserum to SNA was prepared as described below in “*Preparation of anti-mdkCSS antibody*”. A pure *Maackia amurensis* lectin (MAA), which recognizes Siaα2 → 3Gal or GalNAc, were purchased from EY Laboratories, INC. (San Mateo, CA). Mouse anti-lectin antiserum to MAA was prepared as described below in “*Preparation of anti-mdkCSS antibody*”. Anti-Myc monoclonal antibody (mAb.) 9E10 recognizing the Myc epitope (EQKLISEEDLN) was kindly provided by Dr. Gerardy-Schahn (Medizinisches Hochschule Hannover, Germany) and prepared as previously described^38^. The mouse monoclonal anti-His-tag antibody was purchased from Cosmo Bio Co. (Tokyo, Japan). The monoclonal anti-myosin 4 antibody (MF20) was purchased from Thermo Fisher Scientific (Waltham, MA). Anti-β-actin antibody was purchased from Santa Cruz Biotechnology (Dallas, TX). POD-labeled anti-mouse IgG + IgM and anti-rabbit IgG were purchased from American Qualex (San Clemente, CA). Horseradish peroxidase (HRP)-conjugated anti-rabbit IgG and the monoclonal anti-rabbit GAPDH (14C10) antibody were purchased from Cell Signaling Technology (Danvers, MA). Cultured cell lines, Chinese hamster ovary (CHO) cells, were purchased from Riken Cell Bank (Koyadai, Japan). Eight weeks-old BALB/c mice were obtained from Japan SLC (Hamamatsu, Japan).

### Ethics statement and the ARRIVE guidelines

All procedures for the use of animals were approved by the Animal Care and Use Committee of Nagoya University (Permit Number: BBC2019001 for medaka; BBC2019002 for mouse), and performed under the relevant guidelines and regulations, which are set up based on the Animal Research: Reporting of In Vivo Experiments (ARRIVE) guidelines by the same committee.

### Experimental animals

The Nagoya strain of medaka fish, *Oryzias latipes*, was used as the WT. L304Q medaka were provided by NBRP Medaka. To remove the background mutation in L304Q medaka, hetero medaka were mated with Nagoya medaka for over five generations. Fish stocks were maintained in 16-L tanks with a water circulating system at 26 °C, under a 14 h light/10 h dark cycle. The development and phenotype of medaka fish were observed under a microscope (Olympus SZX12 DP80).

### Identification of mutations in the Medaka TILLING library by HRM analysis

The Medaka TILLING library was previously prepared, and contains 5,760 mutant medaka strains induced by ethylnitrosourea (ENU)^23^. Mutations were identified by high-resolution melting curve analysis (HRM analysis) at the National Institute for Basic Biology, as described previously^24^. Exon 6 to exon 7 of the medaka *CSS* gene was amplified using the following primers: 5’-AGGTTCGCATGTCTGTTT-3’(forward);

5’-AGCATATTTATAGAAAACTAGCATGAAGTA-3’(reverse).

### DNA sequencing

Five microliters of PCR products, identified as ENU-induced mutations by HRM assay, were purified using ExoSAP-IT. The purification process was as follows: 5 μL ExoSAP-IT solution was mixed with the PCR product, heated at 37 °C for 15 min, and re-heated at 80 °C for 15 min. One microliter of the purified PCR product was used as a template for sequencing analysis, which was carried out using BigDye Terminator version 3.1 (Applied Biosystems, Foster City, CA) and the ABI 3730xl sequencing platform.

### Genotyping

Fin clips of selected medaka fish or larvae were fixed in 40 μL of methanol and lysed in an appropriate amount of protease K solution (10 mM Tris–HCl (pH 7.5), 10 mM EDTA, and 2 mg/mL proteinase K) prior to heating at 55 °C for 3 h. This was followed by denaturation through protease K activity at 95 °C for 10 min. After centrifugation, the supernatant of each sample was used as genomic DNA. 5’-AGGTTCGCATGTCTGTTT-3’(forward) and 5’-AGCATATTTATAGAAAACTAGCATGAAGTA-3’(reverse) were used to amplify exon 6 and exon 7 of the *CSS* gene by KOD-plus DNA polymerase (Toyobo, Japan), where the L304 mutation was located. Hetero and homo L304Q mutations were identified by HRM assays and confirmed by DNA sequencing analysis.

### Construction of Oryzias latipes CSS plasmids encoding the mutation identified from the TILLING library

The putative medaka *CSS* (mdkCSS) gene or *cmas* was identified in Ensembl (http://asia.ensembl.org/Oryzias_latipes/Info/Index), with transcript ID cmasa-202 (ENSORLG00000018421). The entire open reading frame (ORF) of mdkCSS was amplified from the cDNA of medaka orange red strain utilizing the two primers used to amplify exon 6 and exon 7 of the *CSS* gene during genotyping. The amplified fragment was subsequently cloned with a pGEM-T Easy Vector (Promega, Madison, WI, USA). L304Q, L304I, L304N, L304E, L304A, L304F, L304K, L304S mutations were then generated using the QuikChange Multi Site-Directed Mutagenesis Kit with the primers shown in Table S1.

### Preparation of anti-mdkCSS antibody

The cDNA for the C-domain of mdkCSS (amino acids 1–249) was cloned into a pET32a vector (Invitrogen) and expressed in *Escherichia coli* strain BL21 (DE3) pLysS cells. A single colony of transformed *E. coli* cells was isolated and cultured in 5 mL of Luria broth supplemented with 50 μg/mL of ampicillin (LA), with shaking at 37 °C for 12 h. Thereafter, 5 mL of the culture was inoculated into 500 mL of LA and continuously cultured for 4 h. Once the OD_600_ of the cultured suspension reached 0.5–0.8, 0.4 mM IPTG was added to the culture, which was then incubated at 15 °C for 4 h. Transformant cells were harvested by centrifugation at 6,000 × *g* at 4 °C for 10 min, and suspended in 25 mL of 50 mM Tris–HCl (pH 8.0) prior to sonication. The supernatant obtained from the sonically disrupted cells was mixed with an equal volume of 10 mM imidazole, 1.0 M NaCl, and 50 mM Tris–HCl (pH 8.0), and applied to an Ni^2+^ chelating column (1 × 1.3 cm), which had been equilibrated with 5 mM imidazole, 0.5 M NaCl, and 50 mM Tris–HCl (pH 8.0). After washing with 30 mM imidazole, 0.5 M NaCl, and 50 mM Tris–HCl (pH 8.0), the column was eluted with 100 mM imidazole. The eluted fraction was desalted using VIVA SPIN20 (Sartorius). The thioredoxin tag, S-tag, and His-tag in front of mdkCSS were removed by the recombinant enterokinase and passing through the Ni^2+^ chelating column. The purified C-domain of mdkCSS protein was quantified using bovine serum albumin as a standard by SDS–PAGE/Coomassie Brilliant Blue (CBB) staining. The BALB/c mice was first immunized intraperitoneally with 1 μg of purified C-domain of mdkCSS protein in Freund’s complete adjuvant. Two booster injections were given with 1 μg of purified C-domain of mdkCSS protein in incomplete Freund’s adjuvant at 3-weeks interval, and the antiserum was collected 7 days after injection. The antiserum was affinity-purified with the purified C-domain of mdkCSS-conjugated column prepared using purified C domain of mdkCSS protein immobilized Sepharose 4 beads (Sigma-Aldrich) and used as the anti-mdk-CSS antibody.

### Preparation and detection of recombinant mdkCSS proteins

WT and each mutant mdkCSS cDNA were cloned using a pET32a vector (Invitrogen, USA) and expressed in *E. coli* cells. Five mL of transformant *E. coli* cells was prepared (see above). Transformant cells were harvested by centrifugation at 6,000 × *g* at 4 °C for 10 min, and suspended in 500 μL of 50 mM Tris–HCl (pH 8.0) prior to sonication. The supernatant of sonically disrupted cells was collected by centrifugation at 21,600 × *g* at 4 °C for 15 min and used as the crude enzyme fraction for the enzyme assay. For purification of recombinant enzymes, 5 mL of transformant cells from an overnight culture were incubated in 500 mL of LA at 37 °C for 4 h, followed by induction as described above. The supernatant obtained from the sonically disrupted cells was mixed with an equal volume of 10 mM imidazole, 1.0 M NaCl, and 50 mM Tris–HCl (pH 8.0), and applied to an Ni^2+^ chelating column (1 × 1.3 cm), which had been equilibrated with 5 mM imidazole, 0.5 M NaCl, and 50 mM Tris–HCl (pH 8.0). After washing with 30 mM imidazole, 0.5 M NaCl, and 50 mM Tris–HCl (pH 8.0), the column was eluted with 100 mM imidazole. The eluted fraction was desalted using VIVA SPIN20. SDS–PAGE, Coomassie Brilliant Blue staining, and western blotting were carried out with an anti-mdkCSS antibody (see above) or anti-His antibody.

### Native-PAGE/western blotting

The purified recombinant L304Q and WT CSS proteins (500 ng) were mixed with 2-mercaptoethanol-free sample buffer for electrophoresis, loaded to native-PAGE (6% polyacrylamide gel) and electroblotted onto a PVDF membrane. The PVDF membrane was blocked with 1% skim milk in a phosphate-buffered saline, containing 0.05% Tween-20 (PBST), at 37 °C for 1 h. Thereafter, the membranes were incubated with anti-mdkCSS antibody (diluted 1:1000 ) at 4 °C for 16 h. Peroxidase-conjugated anti-mouse IgG + IgM-POD (0.4 μg/ml) (diluted 1:5000 ) were used as secondary antibodies. Color development was performed using chemiluminescent reagents (GE Healthcare), and the image was acquired by a LuminoGraph II (ATTO. Japan), and the intensity of targeted band was quantified by CS Analyzer4 (ATTO).

### Assay for CMP-Sia synthetase activity

The amount of recombinant protein was determined using the BCA protein assay kit (Pierce, Rockford, IL), with bovine serum albumin concentration serving as the standard. Twenty microliters of recombinant enzyme was added to the pre-mixture, which contained 100 mM Tris–HCl buffer (pH 9.0), 1.0 mM sialic acid (KDN, Neu5Gc, or Neu5Ac), 5.0 mM CTP, 20 mM MgCl_2_, and 0.1 mM Na_3_VO_4_ (which prevents dephosphorylation of CTP). After incubating the reaction mixture at 25 °C for 10 min, excess CTP was digested by adding 25 μL of alkaline phosphatase solution; this contained 30 units of alkaline phosphatase, collected from calf intestines in 200 mM NaOH-glycine (pH 10.0). The reaction mixture was then incubated at 37 °C for 3 h before it was purified by adding 150 μL cold ethanol and centrifuged at 21,600 × *g* for 15 min. Fifty microliter of supernatant was subjected to high-performance liquid chromatography (HPLC) analysis.

HPLC was carried out on a JASCO HPLC system, using a 6.5 × 30 mm resource Q column. The column was eluted at a rate of 1.0 mL/min, with a linear gradient of 0 to 0.2 M NaCl in 20 mM Tris–HCl buffer (pH 8.0) for 10 min. The elution profile was monitored by measuring absorbance at 271 nm, and the peak area was integrated to determine the amount of reaction products. CMP-Neu5Ac was used for quantitative analysis, utilizing a calibrated standard curve, and concentrations of CMP-Neu5Ac, CMP-Neu5Gc, and CMP-KDN in the reaction mixture were calculated using a standard curve. Relative in vitro activity was reported as the amount of CMP-Sia divided by the amount of recombinant enzyme.

### Kinetic analysis

Enzyme concentration, incubation period, and CTP concentration were optimized for kinetic analysis. The former was determined with the BCA protein assay. Briefly, 0.25, 0.5, 1.0, 2.0 and 4.0 mM of Neu5Ac were incubated with 0.2 µg of the purified recombinant mdkCSS protein in 50 µL of 0.1 M Tris–HCl (pH 9.0), which contained 5.0 mM CTP, 20 mM MgCl_2_, and 0.1 mM sodium vanadate, at 25 °C for 15 min. The product was quantified by HPLC, as described above, and kinetic parameters were determined using double-reciprocal Lineweaver–Burk plots. Three independent experiments were performed.

### Thermostability measurement of the recombinant CSS activities

The purified recombinant CSS proteins were pre-heated at 25, 35, or 45 °C for 15 min before in vitro activity measurement. Relative in vitro activity of CSS was reported as the amount of CMP-Neu5Ac divided by the intensity of the recombinant protein, and the value of WT mdkCSS without pre-heating was set to 1.0. Three independent experiments were performed.

### Protein thermal shift assay

The thermal shift assay was conducted according to the manufacturer’s instruction using the Protein Thermal Shift Dye Kit (Cat No. 4461146) and the StepOnePlus Real Time PCR System (Applied Biosystem, Carlsbad, CA). The recombinant L304Q and WT proteins (500 ng) were mixed with the melt reaction buffer containing the fluorescent dye in the wells of the 96-well PCR plate. The plate was heated from 25 to 99 °C with a heating rate of 1 °C/min. The fluorescence intensity was measured with Ex/Em, 409/530 nm.

### Expression of mdkCSS in CHO cells

WT and L304Q mdkCSS cDNA were subcloned into pcDNA4/myc-His A vectors (Invitrogen) using an In-Fusion HD Cloning Kit (Takara Clontech). CHO cells were cultured in MEM-α (Wako, Japan) and supplemented with 100 units/mL penicillin G, 100 μg/mL streptomycin sulfate, and 10% fetal bovine serum (FBS). They were then placed in a 5% CO_2_ and 95% air humidified atmosphere at 37 °C. Thereafter, CHO cells (1.5 × 10^5^) were cultured in a 6-well plate overnight at 37 °C in 5% CO_2_ and transiently transfected with 3 μg of each plasmid using the PEI-Max Transfection Reagent (Polysciences Inc., USA). The expression of CSS proteins was quantified by western blotting using anti-myc antibody.

### SDS-PAGE and western blotting

Cells and tissues were homogenized in phosphate buffered saline (PBS) containing 1% Triton X-100, 1 mM phenylmethylsulfonyl fluoride (PMSF), 50 mM sodium fluoride (NaF), 10 mM β-glycerophosphate, and 1 mM sodium *o*-vanadate. Protease inhibitors were also included (1 μg/mL each of aprotinin, leupeptin, and pepstatin, 2 μg/mL of antipain, and 5 mM EDTA). After centrifugation at 15,000 × *g* for 15 min, at 4 °C, the supernatants were collected and used for further analysis. Each lysate was denatured in Laemmli buffer containing 5% (v/v) β-mercaptoethanol at 60 °C for 20 min. The samples were then electrophoresed using 7.5% or 10% SDS-PAGE and electroblotted onto a PVDF membrane. The following procedures were described in the section “native-PAGE/western blotting” with the exception that blocking was performed with 1% BSA, instead of 1% skim milk.

### Immunostaining

CHO cells were grown on coverslips and transfected with each plasmid using PEI-Max Transfection Reagent (Polysciences Inc., USA). The cells were then fixed with 4% paraformaldehyde at 25 °C for 8 min and permeabilized with 0.2% Triton X-100 in PBS for 15 min for immunostaining. After the cells were blocked with 1% bovine serum albumin (BSA) in PBS for 1 h, myc-tagged CSS was detected through incubation with anti-myc mAb.9E10 (1:500 dilution) at 37 °C for 1 h, and Alexa 488-conjugated anti-mouse IgG (Invitrogen, 1:1000 dilution in PBS) at room temperature for 30 min. For nuclear staining, cells were incubated with 1 μg/mL DAPI at 37 °C for 15 min. Images were obtained using a confocal laser scanning microscope (Olympus BX51).

### RT-PCR

Total RNA was extracted from mdkCSS transient CHO cells using TRI Reagent LS (MRC Inc., Cincinnati, USA). The cDNA of each sample was reverse transcribed using ProtoScript II (NEB, Ipswich, USA), with a random hexamer primer. RT-PCR was performed using the following primers: for mdkCSS gene, 5’-GCATGACTCCGGCTTGTTTG-3’(forward) and.

5’-TCCTTGGAGACTTCCAGGCT -3’(reverse); for β-actin gene,

5’-GTCCCTGTATGCCTCTGGTC-3’(forward) and

5’-GGTAGTCTGTAAGGTCGCGG -3’(reverse).

The amplified fragments were subjected to an agarose-gel electrophoresis and their bands were recorded by UV transilluminator (WEALTEC, Taiwan) and LightCapture software.

### Amount of sialic acid in medaka embryos

The tail tip cut from each medaka embryo was fixed in 40 μL of methanol and subjected to cell lysis in 25 μL of protease K solution (10 mM Tris–HCl [pH 7.5], 10 mM EDTA, and 2 mg/mL proteinase K) at 55 °C for 3 h. This was followed by denaturation of protease K activity at 95 °C for 10 min. After centrifugation, 1 μL of the supernatant was used for genotyping (See above). The remaining medaka embryo was homogenated as above (20 μL) and separated into two equal parts: one part was used for free Sia quantification and the other was used for bound Sia quantification. The free Sia in each sample was quantified using the DMB derivatization method^39^.

### Digital video recording and analysis of the heart contraction

Each embryo in the chorion was immobilized in a hole made by 1.5% agarose. The heart contractions were recorded by a stereomicroscope (Olympus SZX12 DP80). Digital pictures were captured at maximum frame rate at a resolution of 1,360 × 1,024 pixels for up to 3 min and recorded in a PC using CellSens Standard software. Movies of heart movements were processed using ImageJ software. Contraction rhythms were measured based on alterations in the intensity of blood cell flow into and out of ventricle. Regions of interest (ROIs) in the ventricle were selected. The pixel intensities of the ROIs were digitized throughout the entire time series examined using ImageJ software.

### Immunohistochemistry

Medaka, at 14 dpf, were fixed in 4% paraformaldehyde/PBS, embedded in paraffin, and sectioned (each section being 5-μm thick) using a microtome. The sections were deparaffinized in xylene, dehydrated in a graded ethanol series (100, 90, 80, and 70%), incubated in 0.01 M citrated buffer (pH 6.0), and heated in a microwave at 500 W for 1 min (3 times). For lectin staining, sections were blocked in 1% BSA/PBST solution for 1 h and incubated with SNA (1:200) at 4 °C for 16 h. This was followed by incubation with anti-SNA antibodies (1:200) at 4 °C for 16 h, and then Alexa 488-conjugated anti-mouse IgG (Invitrogen, 1:500 dilution in blocking) at 37 °C for 1 h. Images were obtained using a confocal laser scanning microscope (Olympus BX51).

### Statistics

All values were expressed as the mean ± SE (n is three) and p-values were evaluated by the Student’s t-test in Figs. 1–3, and Fig. 7.

## Supplementary Information

Supplementary Information.

## References

[1] Schauer R (2009). Sialic acids as regulators of molecular and cellular interactions. Curr. Opin. Struct. Biol..

[2] Schauer R, Kamerling JP (2018). Exploration of the sialic acid world. Adv. Carbohydr. Chem. Biochem..

[3] Varki NM, Strobert E, Dick EJ, Benirschke K, Varki A (2011). Biomedical differences between human and nonhuman hominids: Potential roles for uniquely human aspects of sialic acid biology. Annu. Rev. Pathol..

[4] Varki A (2017). Are humans prone to autoimmunity? Implications from evolutionary changes in hominin sialic acid biology. J. Autoimmun..

[5] Sato C, Kitajima K (2019). Sialic acids in neurology. Adv. Carbohydr. Chem. Biochem..

[6] Andrews PM (1979). Glomerular epithelial alterations resulting from sialic acid surface coat removal. Kidney Int..

[7] Angata K, Fukuda M (2003). Polysialyltransferases: Major players in polysialic acid synthesis on the neural cell adhesion molecule. Biochimie.

[8] Rutishauser U (2008). Polysialic acid in the plasticity of the developing and adult vertebrate nervous system. Nat. Rev. Neurosci..

[9] Isomura R, Kitajima K, Sato C (2011). Structural and functional impairments of polysialic acid by a mutated polysialyltransferase found in schizophrenia. J. Biol. Chem..

[10] Sato C, Kitajima K (2013). Disialic, oligosialic and polysialic acids: distribution, functions and related disease. J. Biochem..

[11] Abe C, Yi Y, Hane M, Kitajima K, Sato C (2019). Acute stress-induced change in polysialic acid levels mediated by sialidase in mouse brain. Sci. Rep..

[12] Sato C, Kitajima K (2020). Polysialylation and disease. Mol. Aspects Med..

[13] Kean EL (1970). Nuclear cytidine 5'-monophosphosialic acid synthetase. J. Biol. Chem..

[14] Munster AK, Weinhold B, Gotza B, Muhlenhoff M, Frosch M, Gerardy-Schahn R (2002). Nuclear localization signal of murine CMP-Neu5Ac synthetase includes residues required for both nuclear targeting and enzymatic activity. J. Biol. Chem..

[15] Kean EL, Münster-Kühnel AK, Gerardy-Schahn R (2004). CMP-sialic acid synthetase of the nucleus. Biochim. Biophys. Acta.

[16] Mizanur RM, Pohl NL (2008). Bacterial CMP-sialic acid synthetases: Production, properties, and applications. Appl. Microbiol. Biotechnol..

[17] Nakata D, Münster AK, Gerardy-Schahn R, Aoki N, Matsuda T, Kitajima K (2001). Molecular cloning of a unique CMP-sialic acid synthetase that effectively utilizes both deaminoneuraminic acid (KDN) and N-acetylneuraminic acid (Neu5Ac) as substrates. Glycobiology.

[18] Di W, Fujita A, Hamaguchi K, Delannoy P, Sato C, Kitajima K (2017). Diverse subcellular localizations of the insect CMP-sialic acid synthetases. Glycobiology.

[19] Tiralongo J, Fujita A, Sato C, Kitajima K, Lehmann F, Oschlies M, Gerardy-Schahn R, Münster-Kühnel AK (2007). The rainbow trout CMP-sialic acid synthetase utilises a nuclear localization signal different from that identified in the mouse enzyme. Glycobiology.

[20] Oschlies M, Dickmanns A, Haselhorst T, Schaper W, Stummeyer K, Tiralongo J, Weinhold B, Gerardy-Schahn R, von Itzstein M, Ficner R, Münster-Kühnel AK (2009). A C-terminal phosphatase module conserved in vertebrate CMP-sialic acid synthetases provides a tetramerization interface for the physiologically active enzyme. J. Mol. Biol..

[21] Wittbrodt J, Shima A, Schartl M (2002). Medaka–a model organism from the far East. Nat. Rev. Genet..

[22] Iwamatsu T (2011). Chromosome formation during fertilization in eggs of the teleost Oryzias latipes. Methods Mol. Biol..

[23] Taniguchi Y, Takeda S, Furutani-Seiki M, Kamei Y, Todo T, Sasado T, Deguchi T, Kondoh H, Mudde J, Yamazoe M, Hidaka M, Mitani H, Toyoda A, Sakaki Y, Plasterk RH, Cuppen E (2006). Generation of medaka gene knockout models by target-selected mutagenesis. Genome Biol..

[24] Ishikawa T, Kamei Y, Otozai S, Kim J, Sato A, Kuwahara Y, Tanaka M, Deguchi T, Inohara H, Tsujimura T, Todo T (2010). High-resolution melting curve analysis for rapid detection of mutations in a Medaka TILLING library. BMC Mol. Biol..

[25] Kamei Y, Itou J, Oda S, Masui M, Kim JH, Ishikawa T, Yuba S, Kinoshita M, Mitani H, Todo T (2007). Development of a convenient in vitro fertilization method using interspecific hybrids between Oryzias latipes and Oryzias curvinotus. Dev. Growth Differ..

[26] Deng W, Ednie AR, Qi J, Bennett ES (2016). Aberrant sialylation causes dilated cardiomyopathy and stress-induced heart failure. Basic Res. Cardiol..

[27] Taneda Y, Konno S, Makino S, Morioka M, Fukuda K, Imai Y, Kudo A, Kawakami A (2010). Epigenetic control of cardiomyocyte production in response to a stress during the medaka heart development. Dev. Biol..

[28] López-Unzu MA, Durán AC, Soto-Navarrete MT, Sans-Coma V, Fernández B (2019). Differential expression of myosin heavy chain isoforms in cardiac segments of gnathostome vertebrates and its evolutionary implications. Front. Zool..

[29] Varea E, Guirado R, Gilabert-Juan J, Martí U, Castillo-Gomez E, Blasco-Ibáñez JM, Crespo C, Nacher J (2012). Expression of PSA-NCAM and synaptic proteins in the amygdala of psychiatric disorder patients. J. Psychiatr. Res..

[30] Qu R, Sang Q, Wang X, Xu Y, Chen B, Mu J, Zhang Z, Jin L, He L, Wang L (2020). A homozygous mutation in CMAS causes autosomal recessive intellectual disability in a Kazakh family. Ann. Hum. Genet..

[31] Ng BG, Asteggiano CG, Kircher M, Buckingham KJ, Raymond K, Nickerson DA, Shendure J, Bamshad MJ, Ensslen M, Freeze HH, Genomics U, o. W. C. f. M. (2017). Encephalopathy caused by novel mutations in the CMP-sialic acid transporter, SLC35A1. Am. J. Med. Genet. A.

[32] Mori-Yoshimura M, Hayashi YK, Yonemoto N, Nakamura H, Murata M, Takeda S, Nishino I, Kimura E (2014). Nationwide patient registry for GNE myopathy in Japan. Orphanet. J. Rare Dis..

[33] van Karnebeek CD, Bonafé L, Wen XY, Tarailo-Graovac M, Balzano S, Royer-Bertrand B, Ashikov A, Garavelli L, Mammi I, Turolla L, Breen C, Donnai D, Cormier-Daire V, Heron D, Nishimura G, Uchikawa S, Campos-Xavier B, Rossi A, Hennet T, Brand-Arzamendi K, Rozmus J, Harshman K, Stevenson BJ, Girardi E, Superti-Furga G, Dewan T, Collingridge A, Halparin J, Ross CJ, Van Allen MI, Engelke UF, Kluijtmans LA, van der Heeft E, Renkema H, de Brouwer A, Huijben K, Zijlstra F, Heise T, Boltje T, Wasserman WW, Rivolta C, Unger S, Lefeber DJ, Wevers RA, Superti-Furga A (2016). NANS-mediated synthesis of sialic acid is required for brain and skeletal development. Nat. Genet..

[34] Wen XY, Tarailo-Graovac M, Brand-Arzamendi K, Willems A, Rakic B, Huijben K, Da Silva A, Pan X, El-Rass S, Ng R, Selby K, Philip AM, Yun J, Ye XC, Ross CJ, Lehman AM, Zijlstra F, Abu Bakar N, Drögemöller B, Moreland J, Wasserman WW, Vallance H, van Scherpenzeel M, Karbassi F, Hoskings M, Engelke U, de Brouwer A, Wevers RA, Pshezhetsky AV, van Karnebeek CD, Lefeber DJ (2018). Sialic acid catabolism by N-acetylneuraminate pyruvate lyase is essential for muscle function. JCI Insight.

[35] Weinhold B, Sellmeier M, Schaper W, Blume L, Philippens B, Kats E, Bernard U, Galuska SP, Geyer H, Geyer R, Worthmann K, Schiffer M, Groos S, Gerardy-Schahn R, Münster-Kühnel AK (2012). Deficits in sialylation impair podocyte maturation. J. Am. Soc. Nephrol..

[36] Angata T, Matsuda T, Kitajima K (1998). Synthesis of neoglycoconjugates containing deaminated neuraminic acid (KDN) using rat liver alpha2,6-sialyltransferase. Glycobiology.

[37] Sato C, Kitajima K, Inoue S, Seki T, Troy FA, Inoue Y (1995). Characterization of the antigenic specificity of four different anti-(alpha 2–>8-linked polysialic acid) antibodies using lipid-conjugated oligo/polysialic acids. J. Biol. Chem..

[38] Windfuhr M, Manegold A, Muhlenhoff M, Eckhardt M, Gerardy-Schahn R (2000). Molecular defects that cause loss of polysialic acid in the complementation group 2A10. J. Biol. Chem..

[39] Sato C, Inoue S, Matsuda T, Kitajima K (1999). Fluorescent-assisted detection of oligosialyl units in glycoconjugates. Anal. Biochem..

