# Supplementary material for: A point-mutation in the C-domain of CMP-sialic acid synthetase leads to lethality of medaka due to protein insolubility
Source: Sci Rep. 2021 Dec 1;11:23211. doi: 10.1038/s41598-021-01715-3 (PMC8636478; doi:10.1038/s41598-021-01715-3)
Supplement: Supplementary file 1 — Supplementary Information. [file 41598_2021_1715_MOESM1_ESM.pdf]

Supplementary materials

A point-mutation in the C-domain of CMP-sialic acid synthetase leads to lethality of medaka due to protein insolubility

Di Wu[1,2], Hiromu Arakawa[2], Akiko Fujita[2], Hisashi Hashimoto[3], Masahiko Hibi[3], Kiyosi Naruse[4], Yasuhiro Kamei[4,5], Chihiro Sato[1,2], and Ken Kitajima[1,2,*]

[1]Institute of Glyco-core Research, Nagoya University, Chikusa, Nagoya 464-8601, Japan;
[2]Bioscience and Biotechnology Center, and Graduate School of Bioagricultural Sciences, Nagoya University, Chikusa, Nagoya 464-8601, Japan;
[3]Graduate School of Science, Nagoya University, Chikusa, Nagoya, Chikusa, Nagoya 464-8601, Japan;
[4]National Institute of Basic Biology, Nishigonaka 38, Myodaiji, Okazaki 444-8585, Japan;
[5]Department of Basic Biology, School of Life Science, The Graduate University for Advanced Studies, Shonan Village, Hayama, Kanagawa 240-0193, Japan
*Correspondence author: Ken Kitajima E-mail: kitajima@agr.nagoya-u.ac.jp

----

The supplementary information includes 16 figures and 1 table.

Legends for Supplementary Figures:

Supp_FigS1. Alignment of amino acid sequences of medaka CSS (mdkCSS), rainbow trout CSS (rtCSS), mouse CSS (mCSS), and human CSS (hCSS). Identical amino acid residues throughout the four CSSs are colored black, and those in three CSSs are colored gray. The basic clusters assigned as putative nuclear localization signals (NLSs) in mCSS and rtCSS [14,19] are indicated by a black box. The evolutionarily conserved amino acid sequence motifs are double-underlined. Accession: XP_023821652.1 for mdkCSS; NP_001117662.1 for trCSS; NP_034038 for mCSS; and NP_061156.1 for hCSS. The mdkCSS point-mutations identified in the Medaka TILLING library are colored red, and indicated by arrow head. The N-domain and C-domain are separated by a red vertical line.

Supp_FigS2. Genotyping of L304Q medaka. (A) HRM analysis. A fragment containing a mutation at exons 6-7 of the *CSS* gene in each medaka fry was amplified using PCR and examined via HRM assay. The left and right panels show the high-resolution melting curve, and the substitution plots, respectively. The x-axis represents temperature (°C), and the y-axis displays relative signal difference. Red lines indicate the hetero L304Q mutation (L/Q). (B) Nucleotide sequence analysis. The PCR products from homo and WT, indicated by a black line in the HRM assay, were investigated using direct sequencing. Lines in green, red, black, and blue represented adenine, thymine, guanine, and cytosine, respectively. A nucleotide T that was replaced by A in L304Q is shown by a red arrowhead.

Supp_FigS3. Lectin-blotting with MAA for the a2,3-sialic acid epitopes in WT, hetero, and homo medaka of L304Q at 8 dpf and 14 dpf. The whole lysate was applied to SDS-PAGE, followed by the MAA lectin-staining. Immunoblotting of GAPDH was used as a loading control. Full-length blots are presented in Supplementary Figure S12.

Supp_FigS4. Intracellular localization of L304Q and WT mdkCSS in CHO cells. The C-terminally myc-tagged L304Q and WT mdkCSS were transiently expressed in CHO cells. CSS was detected through immunostaining with the anti-myc antibody and the Alexa 488-conjugated anti-mouse IgG (CSS, green). Nuclei were visualized using DAPI (blue). All specimens were observed under a fluorescent microscope. The scale bars indicate 20 µm. (A) Proportions of cell number of normal and abnormal cell shapes. (B) Proportions of cell number with nucleus (N)-, nucleus/cytosol (N/C)-, and cytosol (C)-localized CSS. N, N/C, and C indicate the cells whose CSS is localized in only nucleus, both nucleus and cytosol, and only cytosol, respectively. The bars represent standard deviations from three independent experiments.

Supp_FigS5. Melting curves of L304Q and WT mdkCSS. Purified recombinant L304Q and WT CSSs (500 ng) were subjected to the protein thermal shift (PTS) assay. Normalized melting profiles for are drawn based on the PTS data from StepOneplus™ instrument. The upper 3 curves and the lower 3 curves represent for L304Q and WT, respectively. Tm values are calculated to be: L304Q, 48.6 ± 0.4 °C; L304Q, 45.1 ± 0.6 °C.

Supp_FigS6. Native-PAGE/western blotting of L304Q and WT mdkCSS. The myc-tagged L304Q and WT mdkCSS were expressed in *Escherichia coli*, and purified as a soluble

protein. These recombinant proteins (500 ng) were subjected to the native-PAGE/western blotting without being denatured. For native-PAGE, 6% polyacrylamide gel was used. The recombinant mdkCSS proteins were visualized using anti-mdkCSS antibody.

Supp_FigS7 for Fig_1B. In this experiment, proteins on the SDS-PAGE gel were electronically blotted on the PVDF membrane and immunostained by anti-His antibody. The squared part is used in Fig. 1B.

Supp_FigS8 for Fig_2B. In this experiment, completely the same homogenates were run on two separate polyacrylamide gels. The gels were blotted on the PVDF membranes and subjected to immunoblotting using SNA lectin (upper panel) or anti-β-actin antibody (lower panel) as the primary probes. The squared parts are used in Fig. 2B.

Supp_FigS9 for Fig_2C. In this experiment, a large PVDF membrane, after electronically blotted, was cut into the upper and lower parts according to the molecular weight, followed by immunostaining with 12E3 for polysialic acid (upper panel) and with anti-β-actin for beta-actin (lower panel), respectively. The squared parts are used in Fig. 2C.

Supp_FigS10 for Fig_3C. In this experiment, a large PVDF membrane, after electronically blotted from one gel, was cut into the upper and lower parts according to the molecular weight, followed by immunostaining with myosinHC for myosin heavy chain (upper panel) and with anti-GAPDH for GAPDH (lower panel), respectively. The squared parts are used in Fig. 3C.

Supp_FigS11 for Fig_5A. In this experiment, proteins on the SDS-PAGE gel were electronically blotted on the PVDF membrane and immunostained by anti-CSS antibody. The squared part is used in Fig. 5A.

Supp_FigS12 for Fig_6A. In this experiment, a large PVDF membrane, after electronically blotted from one gel, was cut into the upper and lower parts according to the molecular weight, followed by immunostaining with anti-myc for mdkCSS (upper panel) and with anti-GAPDH for GAPDH (lower panel), respectively. The squared parts are used in Fig. 6A.

Supp_FigS13 for Fig_6B. Agarose gel electrophoresis profiles of the amplified fragments by the mdkCSS- or b-actin-specific primers using the primary cDNAs from WT- and L304Q-expressing CHO cells. Upper panel, the results of mdkCSS-specific fragments; Lower panel, the results of β-actin-specific fragments. The squared parts are used in Fig. 6B.

Supp_FigS14 for Fig_7A. In this experiment, proteins on the SDS-PAGE gel were electronically blotted on the PVDF membrane and immunostained by anti-His antibody. The squared parts are used in Fig. 7A.

Supp_FigS15 for Supp_FigS3. In this experiment, completely the same homogenates were run on two separate polyacrylamide gels. The gels were blotted on the PVDF membranes and subjected to immunoblotting using MAA lectin (upper panel) or anti-GAPDH antibody (lower panel) as the primary probes. The squared parts are used in Supp_FigS3.

Supp_FigS16 for Supp_FigS6. In this experiment, proteins on the native-PAGE gel were electronically blotted on the PVDF membrane and immunostained by anti-mdkCSS antibody. The left side-slit was cut and CBB-stained to visualized the molecular weight markers for analyzing the right side membrane. The squared parts are used in Supp_FigS6.

Table S1. Oligonucleotide primers used to construct the plasmids for the expression of various L304 mutations

Primer name	Oligonucleotide Sequence (5'-3')
L304Q-forward	GTGGAGGTGGTTCTACAGAACTGCAGTCAGGAC
L304Q-reverse	GTCCTGACTGCAGTTCTGTAGAACCACCTCCAC
L304I-forward	GGTTCTAATTAACTGCAGTCAGGAC
L304I-reverse	CTGACTGCAGTTAATTAGAACCACC
L304N-forward	GGTTCTAAACAACTGCAGTCAGGAC
L304N-reverse	CTGACTGCAGTTGTTTAGAACCACC
L304E-forward	GGTGGTTCTAGAAAACTGCAGTCAG
L304E-reverse	CTGACTGCAGTTTTCTAGAACCACC
L304A-forward	GGTGGTTCTAGCGAACTGCAGTCAG
L304A-reverse	CTGACTGCAGTTCGCTAGAACCACC
L304F-forward	GGTGGTTCTATTCAACTGCAGTCAG -
L304F-reverse	CTGACTGCAGTTGAATAGAACCACC
L304K-forward	GGTGGTTCTAAAGAACTGCAGTCAG
L304K-reverse	GCAGTTCTTTAGAACCACCTCCACC
L304S-forward	GGTTCTATCTAACTGCAGTCAGGAC
L304S-reverse	GACTGCAGTTAGATAGAACCACCTC

Supp_Fig. S1

```
mdkCSS    1 --------------------MSSPKQPLDEEQGGPHMKKARGRHVAALILARGGSKGIPL  40
rtCSS     1 -------------MAAAKKRTQSDIEDVRDRKAKVIKDSGEKRHIAALILARGGSKGIPL  47
hCSS      1 MDSVEKGAATSVSNPRGRPSRGRPPKLQRNSRGGQGRGVEKPPHLAALILARGGSKGIPL  60
mCSS      1 MDALEKGAATSGPAPRGRPSRGRPPKLQR-SRGA-GRGLEKPPHLAALVLARGGSKGIPL  58

mdkCSS   41 KNIKKLAGVPLIGWVLRAAHDSGLFDSVWVSTDHDEIAKVAESFEAKVHRRSLEVSKDSS 100
rtCSS    48 KNIKVLAGVPLIGWVLRAAVDSKQFDSVWVSTDHDDIEKVAKTWGAQVHRRSPEVSKDSS 107
hCSS     61 KNIKHLAGVPLIGWVLRAALDSGAFQSVWVSTDHDEIENVAKQFGAQVHRRSSEVSKDSS 120
mCSS     59 KNIKRLAGVPLIGWVLRAALDAGVFQSVWVSTDHDEIENVAKQFGAQVHRRSSETSKDSS 118

mdkCSS  101 TSLEAIQEFVKDHPDVDVVCNIQATSPCLHPDHLSEALKLITEDGYDSVFSVVRRHQFRW 160
rtCSS   108 SSLDTIQEFARLNPEVDVICHIQATSPCLHPFHLKEALEMITKQGFTSVFSVVRRHHFRW 167
hCSS    121 TSLDAIIEFLNYHNEVDIVGNIQATSPCLHPTDLQKVAEMIREEGYDSVFSVVRRHQFRW 180
mCSS    119 TSLDAIVEFLNYHNEVDIVGNIQATSPCLHPTDLQKVAEMIREEGYDSVFSVVRRHQFRW 178

mdkCSS  161 KEVKKGGSEVTVPLNLDPKNRPRRQDWDGELVENGSFYFATTSLIEKGLLQGGKMAYYEM 220
rtCSS   168 QEVKKGGSVATQPLNLDPQNRPRRQDWDGELCENGSFYIYTRATIERGL-QGGKWAYYEM 226
hCSS    181 SEIQKGVREVTEPLNLNPAKRPRRQDWDGELYENGSFYFAKRHLIEMGYLQGGKMAYYEM 240
mCSS    179 SEIQKGVREVTEPLNLNPAKRPRRQDWDGELYENGSFYFAKRHLIEMGYLQGGKMAYYEM 238

mdkCSS  221 EPQYSIDIDIDIDWPVAEQRVLRYGYFGKVPPEVVRLMFCKLSGCLTDGRITISSSEKEM 280
rtCSS   227 LPEYSVDIDVDIDWPVAEQRVLRFGYFGLDKPEVVRLLLCNVSGCLTDGRVLISVSGEEM 286
hCSS    241 RAEHSVDIDVDIDWPIAEQRVLRYGYFGKEKLKEIKLLVCNIDGCLTNGHIYVSGDQKEI 300
mCSS    239 RAEHSVDIDVDIDWPIAEQRVLRFGYFGKEKLKEIKLLVCNIDGCLTNGHIYVSGDQKEI 298

mdkCSS  281 VSFHIRDMEGLRMLKSEKVEVVLLNCSQDPIGKSLVEKLRERTGCQVLTVGKKPLEDLQP 340
rtCSS   287 VSVNTRDTMGIRMLQREGVEVILISSSEDLLTKALADNLSQRTGCEVRQLGKDIQGEVIA 346
hCSS    301 ISYDVKDAIGISLLKKSGIEVRLI--SERACSKQTLSSL--KLDCKMEVSVSDKLAVVDE 356
mCSS    299 ISYDVKDAIGISLLKKSGIEVRLI--SERACSKQTLSAL--KLDCKTEVSVSDKLATVDE 354

mdkCSS  341 FLKKKKLEWKDVAYMGNDGADADCLNQSGLSAVPGDAPAEAVKAAKYSCHHTTGNGAVRE 400
rtCSS   347 MMDDKDLDWKEVAYMGNDAPDVDCLNLAGLSAVPRDAPVVAINAAKYSCHSAAGLGAVRE 406
hCSS    357 WRKEMGLCWKEVAYLGNEVSDEECLKRVGLSGAPADACSTAQKAVGYICKCNGGRGAIRE 416
mCSS    355 WRKEMGLCWKEVAYLGNEVSDEECLKRVGLSAVPADACSGAQKAVGYICKCSGGRGAIRE 414

mdkCSS  401 FAEYIVRKKQEAMSKKQQVPADGLDSGYVTNSSGSQSESEFSLSGEAINYCGVLQHPR   458
rtCSS   407 FSEHILLLKKKAKSQMEQDRIHRNTF--------------------------------   432
hCSS    417 FAEHICLLMEKVNNSCQK----------------------------------------   434
mCSS    415 FAEHIFLLIEKVNNSCQK----------------------------------------   432
```

Supp_Fig. S1. Alignment of amino acid sequences of medaka CSS (mdkCSS), rainbow trout CSS (rtCSS), mouse CSS (mCSS), and human CSS (hCSS). Identical amino acid residues throughout the four CSSs are colored black, and those in three CSSs are colored gray. The basic clusters assigned as putative nuclear localization signals (NLSs) in mCSS and rtCSS [14,19] are indicated by a black box. The evolutionarily conserved amino acid sequence motifs are double-underlined. Accession: XP_023821652.1 for mdkCSS; NP_001117662.1 for trCSS; NP_034038 for mCSS; and NP_061156.1 for hCSS. The mdkCSS point-mutations identified in the Medaka TILLING library are colored red, and indicated by arrow head. The N-domain and C-domain are separated by a red vertical line.

Supp_FigS2

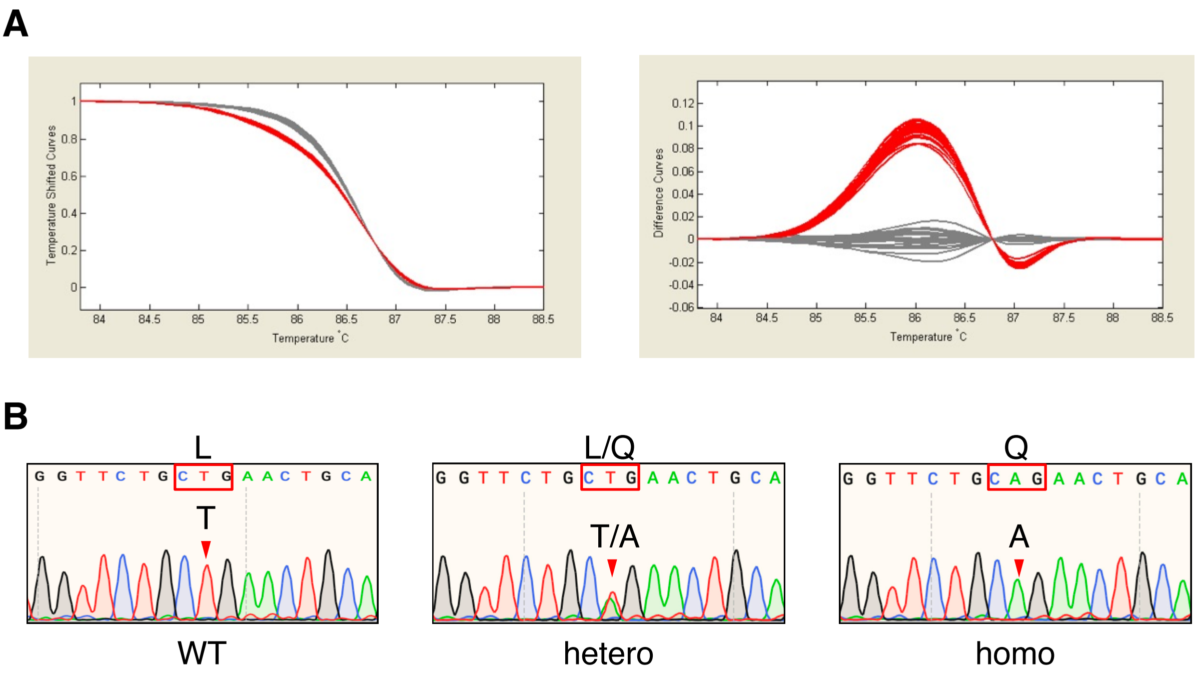

Supp_FigS2 Genotyping of L304Q medaka. (A) HRM analysis. A fragment containing a mutation at exons 6-7 of the *CSS* gene in each medaka fry was amplified using PCR and examined via HRM assay. The left and right panels show the high-resolution melting curve, and the substitution plots, respectively. The x-axis represents temperature (°C), and the y-axis displays relative signal difference. Red lines indicate the hetero L304Q mutation (L/Q). (B) Nucleotide sequence analysis. The PCR products from homo and WT, indicated by a black line in the HRM assay, were investigated using direct sequencing. Lines in green, red, black, and blue represented adenine, thymine, guanine, and cytosine, respectively. A nucleotide T that was replaced by A in L304Q is shown by a red arrowhead.

Supp_FigS3

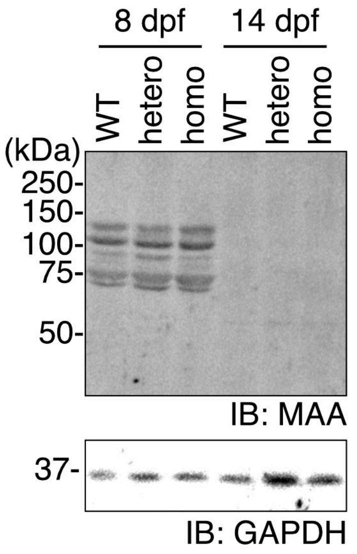

Supp_FigS3 Lectin-blotting with MAA for the α2,3-sialic acid epitopes in WT, hetero, and homo medaka of L304Q at 8 dpf and 14 dpf. The whole lysate was applied to SDS-PAGE, followed by the MAA lectin-staining. Immunoblotting of GAPDH was used as a loading control. Full-length blots are presented in Supplementary Figure S12.

Supp_FigS4

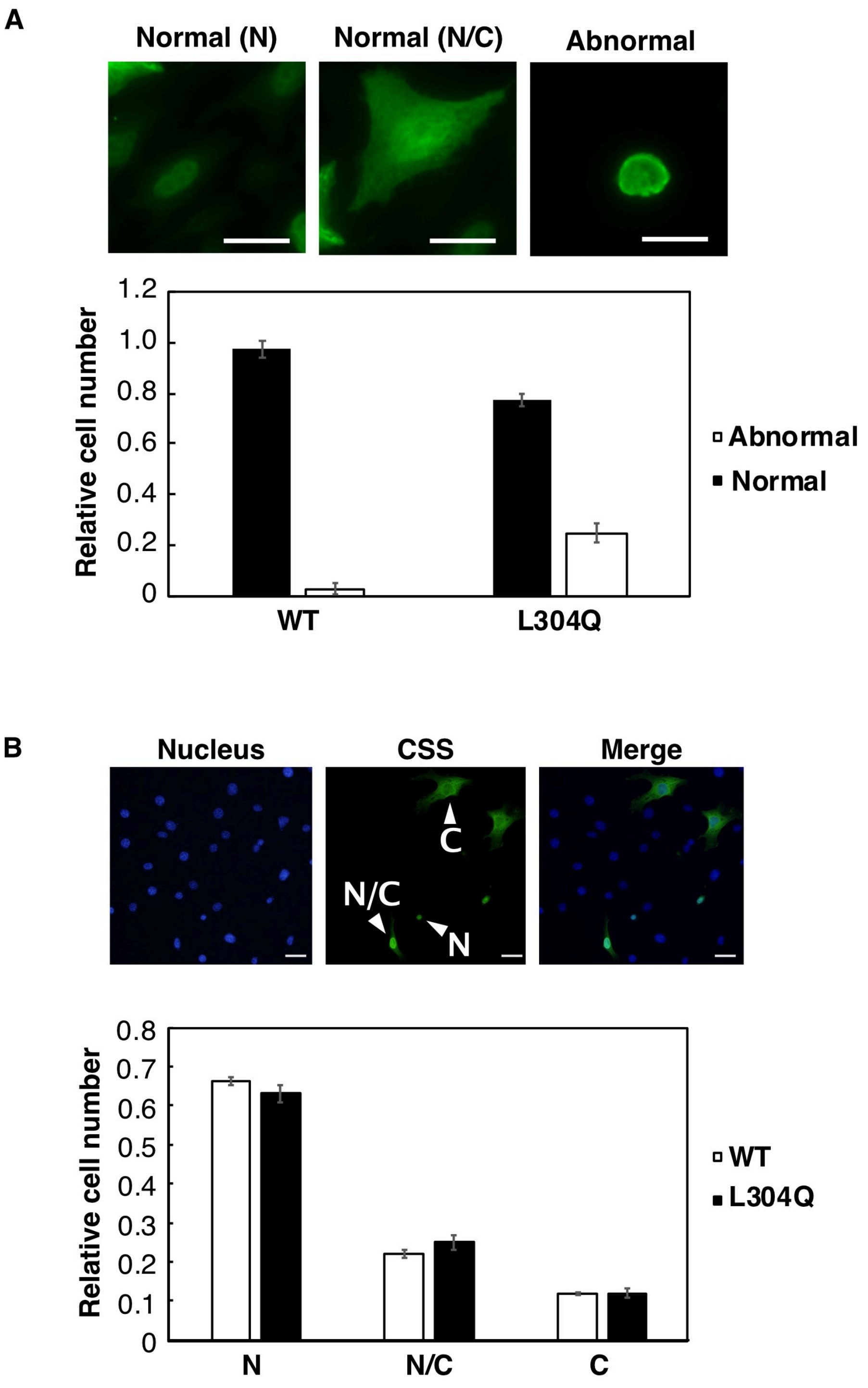

Supp_FigS4. Intracellular localization of L304Q and WT mdkCSS in CHO cells. The C-terminally myc-tagged L304Q and mdkCSS-WT were transiently expressed in CHO cells. CSS was detected through immunostaining with the anti-myc antibody and the Alexa 488-conjugated anti-mouse IgG (CSS, green). Nuclei were visualized using DAPI (blue). All specimens were observed under a fluorescent microscope. The scale bars indicate 20 μm. (A) Proportions of cell number of normal and abnormal cell shapes. (B) Proportions of cell number with nucleus (N)-, nucleus/cytosol (N/C)-, and cytosol (C)-localized CSS. N, N/C, and C indicate the cells whose CSS is localized in only nucleus, both nucleus and cytosol, and only cytosol, respectively. The bars represent standard deviations from three independent experiments.

Supp_FigS5

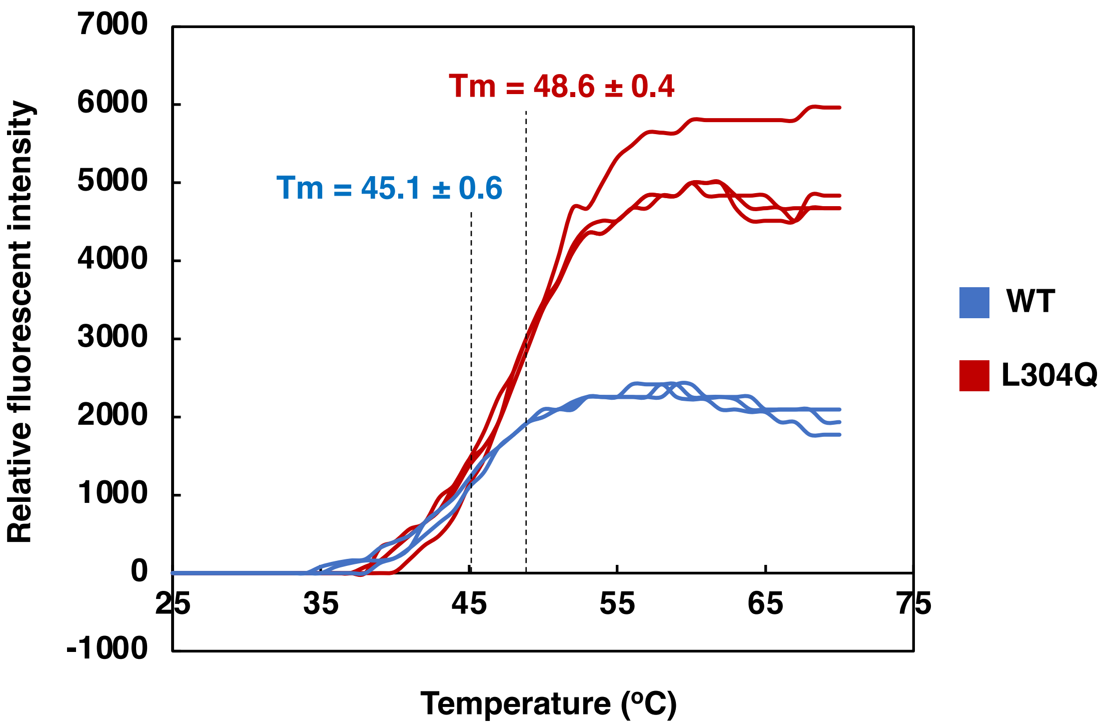

Supp_FigS5. Melting curves of L304Q and WT mdkCSS. Purified recombinant L304Q and WT CSSs (500 ng) were subjected to the protein thermal shift (PTS) assay. Normalized melting profiles for are drawn based on the PTS data from StepOneplus™ instrument. The upper 3 curves and the lower 3 curves represent for L304Q and WT, respectively. Tm values are calculated to be: L304Q, 48.6 ± 0.4 °C; L304Q, 45.1 ± 0.6 °C.

Supp_FigS6

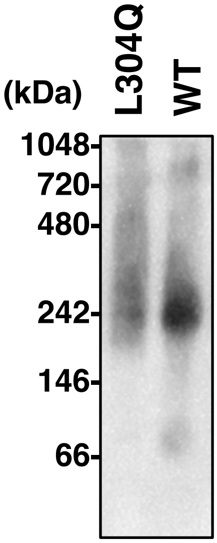

Supp_FigS6. Native-PAGE/western blotting of L304Q and WT mdkCSS. The myc-tagged L304Q and WT mdkCSS were expressed in *Escherichia coli*, and purified as a soluble protein. These recombinant proteins (500 ng) were subjected to the native-PAGE/western blotting without being denatured. For native-PAGE, 6% polyacrylamide gel was used. The recombinant mdkCSS proteins were visualized using anti-mdkCSS antibody.

Supp_FigS7 for Fig_1B

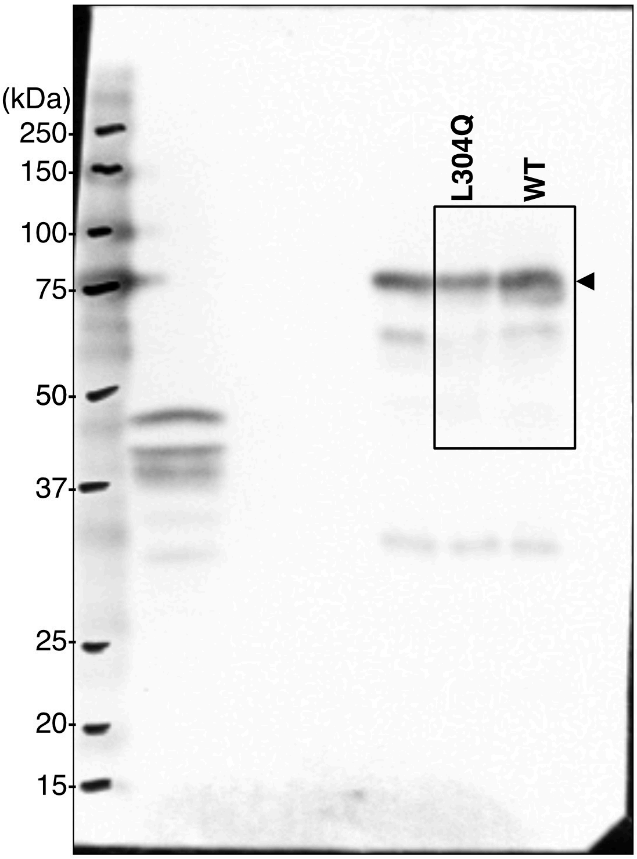

IB: anti-His tag

Supp_FigS7 for Fig_1B. In this experiment, proteins on the SDS-PAGE gel were electronically blotted on the PVDF membrane and immunostained by anti-His antibody. The squared part is used in Fig. 1B.

Supp_FigS8 for Fig_2B

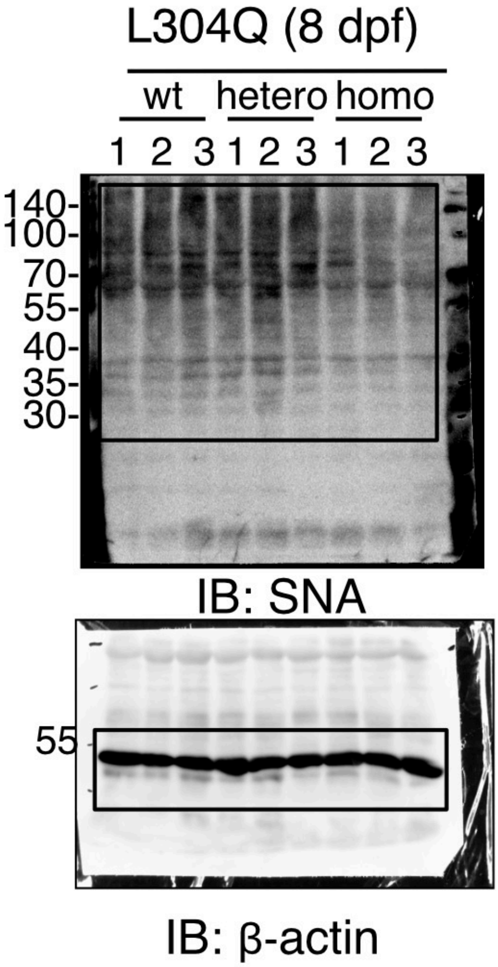

Supp_FigS8 for Fig_2B In this experiment, completely the same homogenates were run on two separate polyacrylamide gels. The gels were blotted on the PVDF membranes and subjected to immunoblotting using SNA lectin (upper panel) or anti-β-actin antibody (lower panel) as the primary probes. The squared parts are used in Fig. 2C.

Supp_FigS9 for Fig_2C

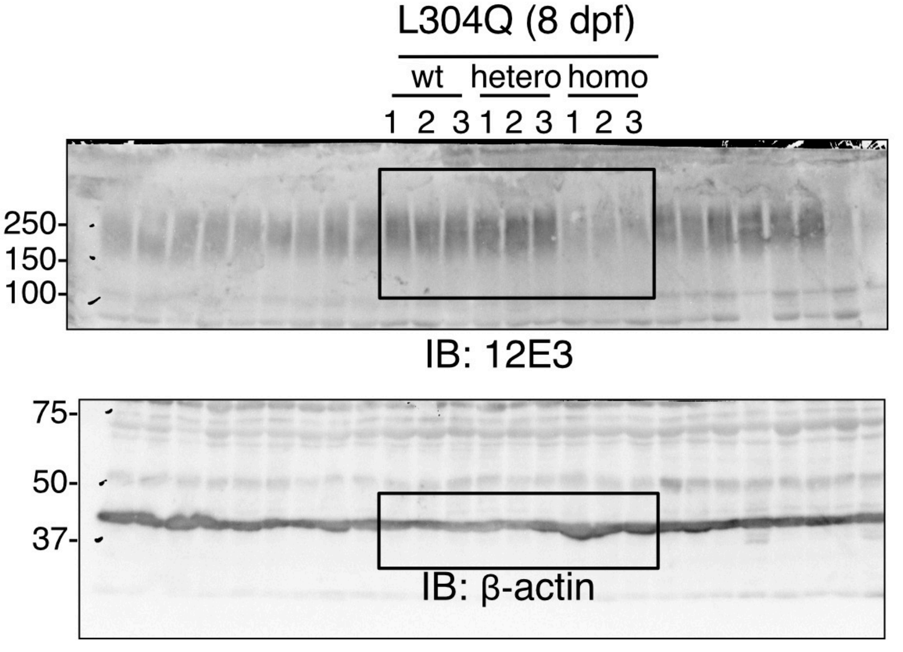

Supp_FigS9 for Fig_2C In this experiment, a large PVDF membrane, after electronically blotted, was cut into the upper and lower parts according to the molecular weight, followed by immunostaining with 12E3 for polysialic acid (upper panel) and with anti-β-actin for beta-actin (lower panel), respectively. The squared parts are used in Fig. 2B.

Supp_FigS10 for Fig_3C

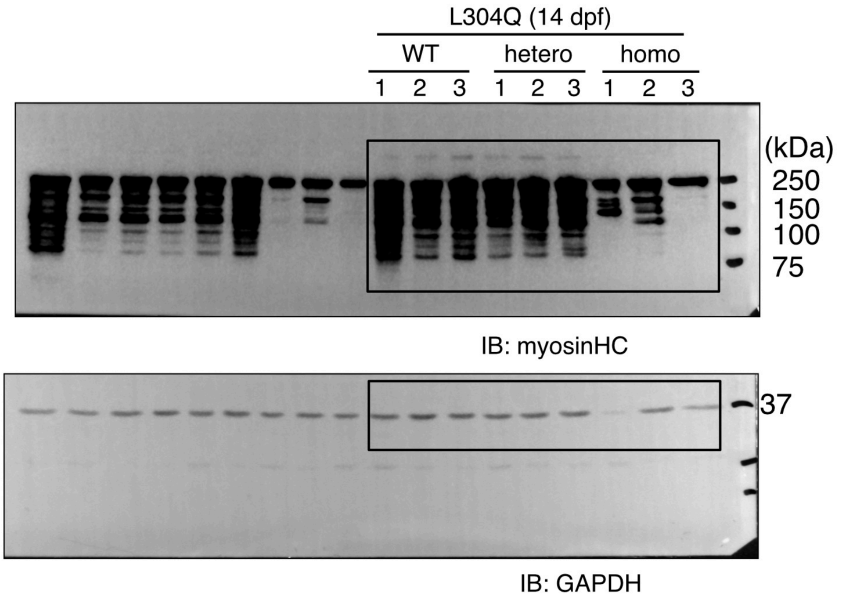

Supp_FigS10 for Fig_3C In this experiment, a large PVDF membrane, after electronically blotted from one gel, was cut into the upper and lower parts according to the molecular weight, followed by immunostaining with myosinHC for myosin heavy chain (upper panel) and with anti-GAPDH for GAPDH (lower panel), respectively. The squared parts are used in Fig. 3C.

Supp_FigS11 for Fig_5A

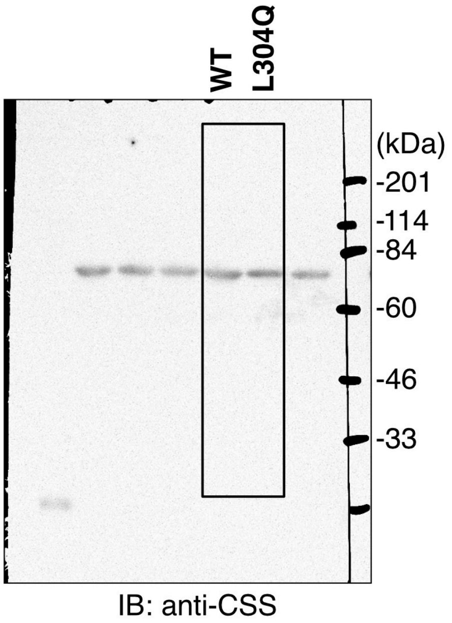

Supp_FigS11 for Fig_5A. In this experiment, proteins on the SDS-PAGE gel were electronically blotted on the PVDF membrane and immunostained by anti-CSS antibody. The squared part is used in Fig. 5A.

Supp_FigS12 for Fig_6A

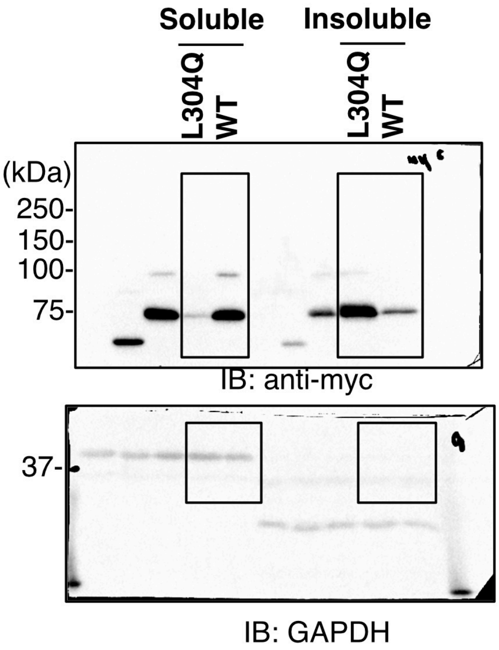

Supp_FigS12 for Fig_6A. In this experiment, a large PVDF membrane, after electronically blotted from one gel, was cut into the upper and lower parts according to the molecular weight, followed by immunostaining with anti-myc for mdkCSS (upper panel) and with anti-GAPDH for GAPDH (lower panel), respectively. The squared parts are used in Fig. 6A.

Supp_FigS13 for Fig_6B

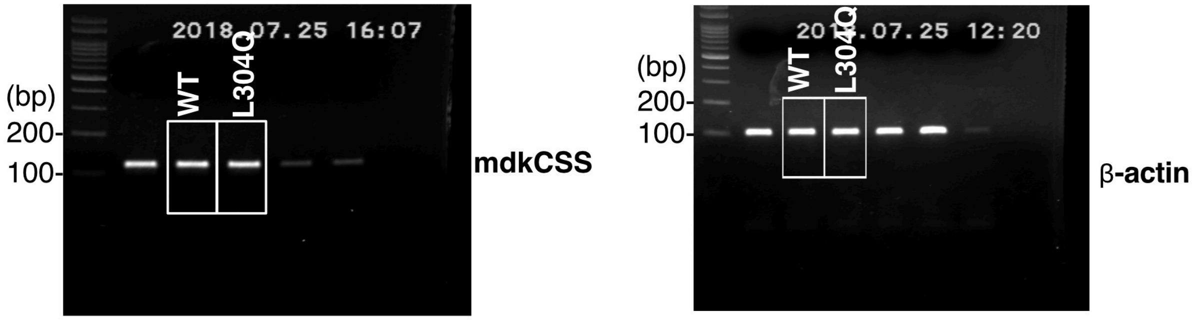

Supp_FigS13 for Fig_6B. Agarose gel electrophoresis profiles of the amplified fragments by the mdkCSS- or β-actin-specific primers using the primary cDNAs from WT- and L304Q-expressing CHO cells. Upper panel, the results of mdkCSS-specific fragments; Lower panel, the results of β-actin-specific fragments. The squared parts are used in Fig. 6B.

Supp_FigS14 for Fig_7A

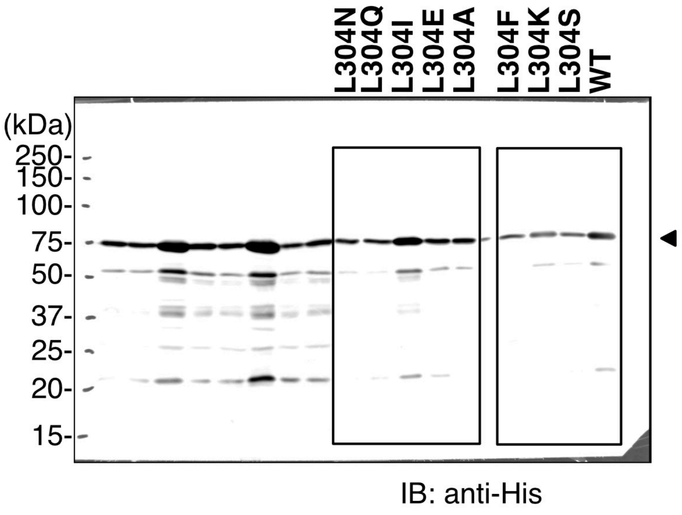

Supp_FigS14 for Fig_7A. In this experiment, proteins on the SDS-PAGE gel were electronically blotted on the PVDF membrane and immunostained by anti-His antibody. The squared parts are used in Fig. 7A.

Supp_FigS15 for Supp_FigS3

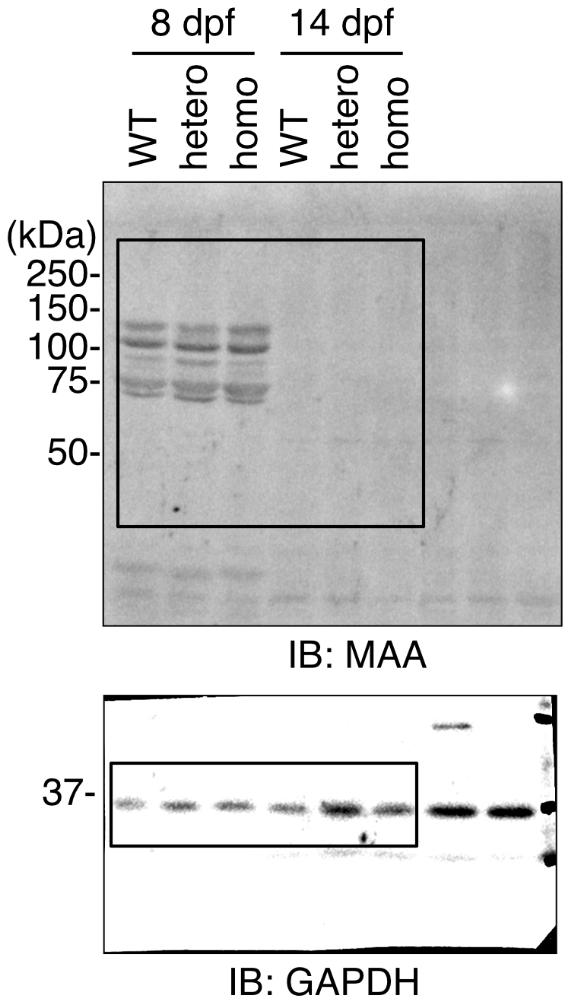

Supp_FigS15 for Supp_FigS3. In this experiment, completely the same homogenates were run on two separate polyacrylamide gels. The gels were blotted on the PVDF membranes and subjected to immunoblotting using MAA lectin (upper panel) or anti-GAPDH antibody (lower panel) as the primary probes. The squared parts are used in Supp_FigS3.

Supp_FigS16 for Supp_FigS6

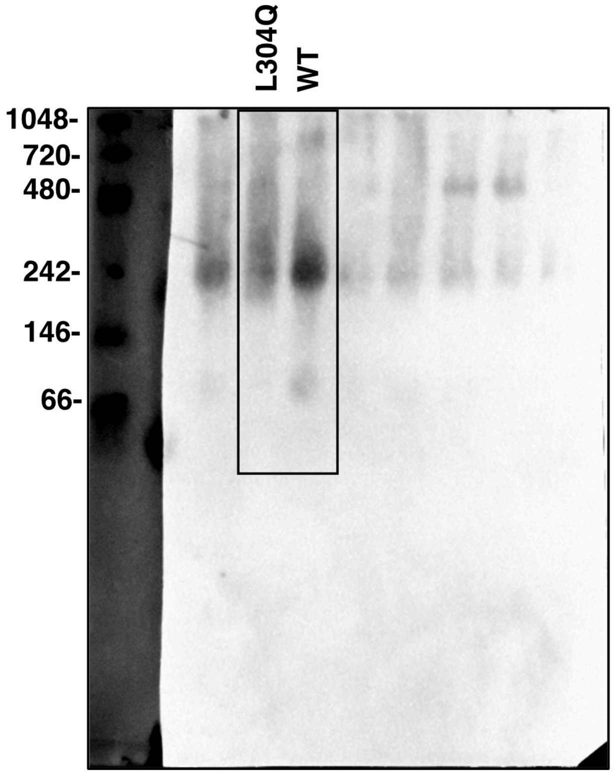

Supp_FigS16 for Supp_FigS6. In this experiment, proteins on the native-PAGE gel were electronically blotted on the PVDF membrane and immunostained by anti-mdkCSS antibody. The left side-slit was cut and CBB-stained to visualized the molecular weight markers for analyzing the right side membrane. The squared parts are used in Supp_FigS6.
